# Physiological and genetic convergence supports hypoxia resistance in high-altitude songbirds

**DOI:** 10.1371/journal.pgen.1009270

**Published:** 2020-12-28

**Authors:** Ying Xiong, Liqing Fan, Yan Hao, Yalin Cheng, Yongbin Chang, Jing Wang, Haiyan Lin, Gang Song, Yanhua Qu, Fumin Lei

**Affiliations:** 1 Key Laboratory of Zoological Systematics and Evolution, Institute of Zoology, Chinese Academy of Sciences, Beijing, China; 2 University of Chinese Academy of Sciences, Beijing, China; 3 National Forest Ecosystem Observation & Research Station of Nyingchi Tibet, Institute of Plateau Ecology, Tibet Agriculture & Animal Husbandry University, Linzhi City, China; 4 Key Laboratory of Forest Ecology in Tibet Plateau (Tibet Agriculture & Animal Husbandry University), Ministry of Education, Linzhi City, China; 5 Center for Excellence in Animal Evolution and Genetics, Chinese Academy of Sciences, Kunming, China; University of Wisconsin–Madison, UNITED STATES

## Abstract

Skeletal muscle plays a central role in regulating glucose uptake and body metabolism; however, highland hypoxia is a severe challenge to aerobic metabolism in small endotherms. Therefore, understanding the physiological and genetic convergence of muscle hypoxia tolerance has a potential broad range of medical implications. Here we report and experimentally validate a common physiological mechanism across multiple high-altitude songbirds that improvement in insulin sensitivity contributes to glucose homeostasis, low oxygen consumption, and relative activity, and thus increases body weight. By contrast, low-altitude songbirds exhibit muscle loss, glucose intolerance, and increase energy expenditures under hypoxia. This adaptive mechanism is attributable to convergent missense mutations in the *BNIP3L* gene, and *METTL8* gene that activates *MEF2C* expression in highlanders, which in turn increases hypoxia tolerance. Together, our findings from wild high-altitude songbirds suggest convergent physiological and genetic mechanisms of skeletal muscle in hypoxia resistance, which highlights the potentially medical implications of hypoxia-related metabolic diseases.

## Author summary

We integrate physiological, genomic and functional experimental methods to illustrate the convergent molecular and genetic mechanisms of hypoxia resistance in wild songbirds. We find that highland native songbirds have greater body and muscle mass with an increase in insulin sensitivity and aerobic metabolism comparing to lowland songbirds. Comparative transcriptomes and functional experiments show that *MEF2C* is a shared overexpression gene in highland songbirds and maintains muscle mass and insulin sensitivity in hypoxia. Genetically, four genes with high genetic divergence between altitudinal tree sparrows have convergent nonsynonymous substitutions in snow finches and three of these are related with hypoxia including *HBB*, *BNIP3L* and *METTL8*. *METTL8* may regulate the expression of *MEF2C* and thus increase muscle hypoxia resistance. These results uncover the physiological and genetic convergence on muscle hypoxia resistance in highland songbirds, and support novel and valuable insights into highland adaptation.

## Introduction

Hypoxia, including ambient hypoxia (high-altitude exposure) [1] and pathological hypoxia (diseases) [2], induces metabolic alterations, muscle loss and insulin resistance [3–5]. At high altitude, hypoxia resulting from the low barometric pressure is a significantly severe challenge to aerobic exercise and thermogenesis in small endotherms, because a high rate of O_2_ flux should be concurrently sustained to thermogenesis in cold temperature [6]. Skeletal muscle plays a central role in regulating glucose uptake and body metabolism [7]. Therefore, muscle physiological modifications, including typically greater capillary density, more oxidative fiber, and higher proportion of subsarcolemmal mitochondria have been found in highland animals [6, 8, 9]. However, the physiological and genetic changes in skeletal muscles supporting hypoxia resistance are poorly understood, particularly for high-altitude songbirds.

In evolutionary biology, a growing body of literature has shown convergent physiological and genetic adaptations to high elevations across variant species. For example, high-altitude mice have evolved a physiological strategy of increasing carbohydrate utilization to economize oxygen use [10]. Convergent gene expression and genetic mechanisms for hypoxia adaptation have been elucidated in a number of mammals [11], birds [12], reptiles [13], and amphibians [14]. Generally, similar phenotypes occurring in different species under the same environmental stressors are proposed to depend on common physiological and genetic mechanisms. However, most physiological convergence lacks genetic evidence or genetic convergence is confirmed merely at the protein or cellular levels *in vitro* [10, 13]. To date, whether convergent gene expression shifts or mutants for hypoxia adaptation indeed drive a similar physiology change and subsequently a morphological phenotype in whole organisms remains unclear.

Here we mainly focused on snowfinches (*Onychostruthus taczanowskii*, and *Pyrgilauda ruficollis*) and tree sparrow (*Passer montanus*) belonging to the Old World sparrows (Passeridae). Snowfinches are endemic natives with a strict elevation distribution ranging from 3,500 to 5,100m on the Qinghai-Tibet Plateau (QTP), whereas tree sparrows are an introduced colonizer on the QTP despite its altitudinal distribution from sea level to 4,400m [15, 16]. We integrated behavioral, physiological and transcriptomic data across altitudinal populations and species to chiefly reveal common physiological and genetic mechanisms of muscle hypoxia tolerance. We report and experimentally validate using RNA interference *in vivo* a convergent mechanism that improves insulin sensitivity based on natural selection of standing genetic variations are essential to muscle hypoxia resistance in high-altitude songbirds, which provides novel and valuable insights into understanding vertebrate hypoxia adaptation.

## Results

### Muscle mass and phenotype variation in high-altitude songbirds

**Fig 1:**
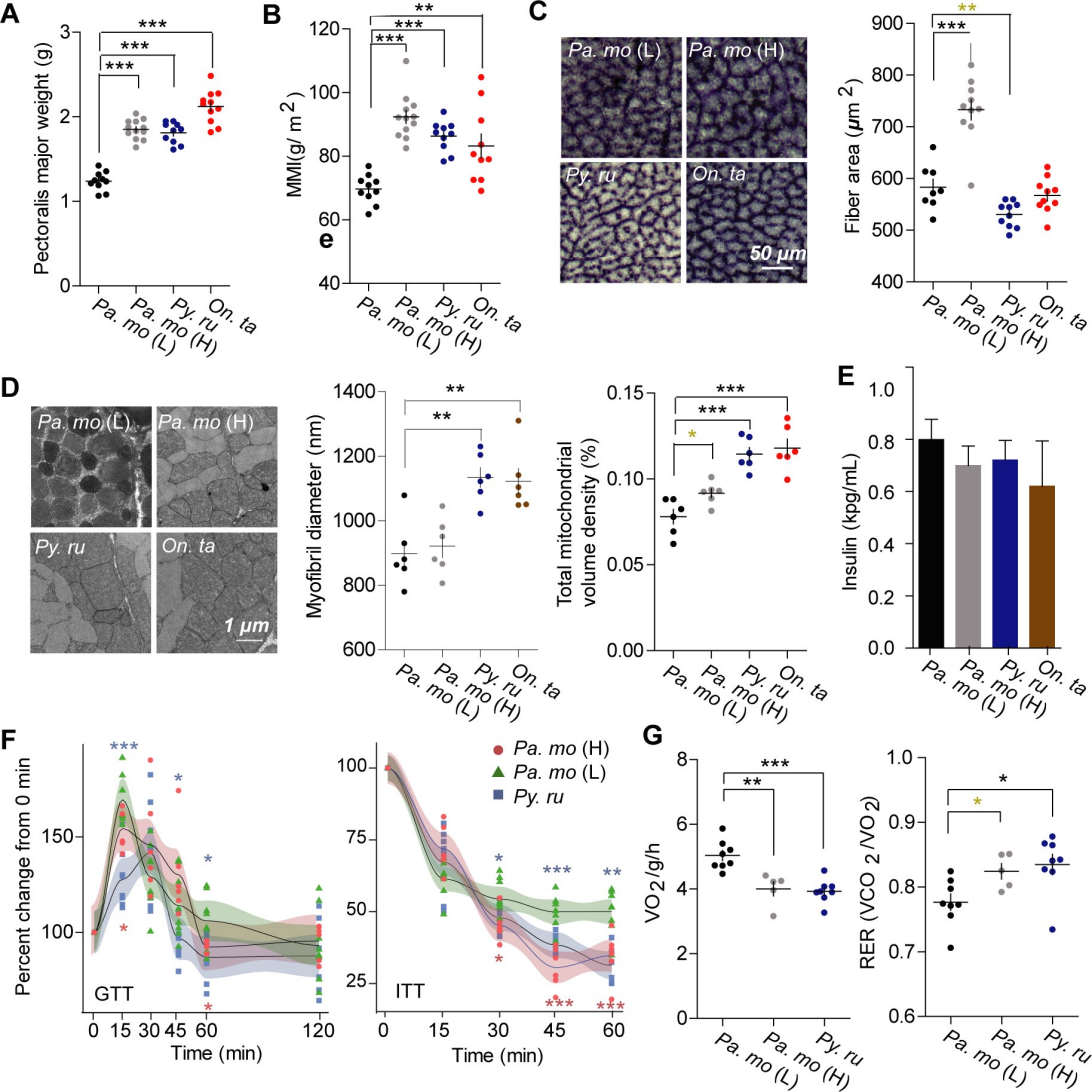
Increase in muscle mass and insulin sensitivity in high-altitude songbirds. (A) High-altitude birds increase pectoralis major weight [n = 10 for *Pa*. *mo* (H) and snow finches, and 12 for *Pa*. *mo* (L)]. (B) Muscle mass *vs*. body length ratio (MMI) was also greater in highland birds. Succinate dehydrogenase (SDH) staining (C) and transmission electron microscopy (TEM) (D) images of pectoralis muscle show variable fiber size or myofibril size, and a significantly greater mitochondrial volume densities in highland songbirds [n = 8 for *Pa*. *mo* (L), and 10 for highland birds for SDH; n = 6 each for TEM]. (E) Fasting plasma insulin was similar across altitudinal birds (n = 7 each). (F) Glucose and insulin tolerance test confirmed the improvement in insulin sensitivity in high-altitude birds [n = 6 each for insulin measurement; n = 8 for *Pa*. *mo* (L) and *Py*. *ru*, and 6 for *Pa*. *mo* (H) for GTT and ITT tests]. (G) Lower metabolic rates and higher carbohydrate utilization were observed in highlanders [n = 8 for *Pa*. *mo* (L) and *Py*. *ru*, and 5 for *Pa*. *mo* (H)].

We found a tendency that body mass increases with the elevation in snow finches and tree sparrow (*p* value <0.05) (S1 Fig and S1 Data). And high-altitude songbirds have greater pectoralis muscle mass compared to lowlanders (*p* values <0.05) (Fig 1A). To exclude the influence of body size, we compared their muscle mass index (MMI, calculated as muscle mass/ body length^2^), which is also greater in highlanders (*p* values <0.05) (Fig 1B). Therefore, for highland songbirds, skeletal muscle is an important contributor to the increase in body weight.

Considering muscle fiber-type transition when ascending to high altitudes in mammals [17] and a greater numerical density of oxidative fibres in high-altitude deer mice [18], we further compared muscle phenotypic variations among altitudinal species and populations. Histological examination revealed that the pectoralis muscles of all birds contained only fast oxidative glycolytic fibers. Despite the homogeneous fiber-type composition, highland tree sparrows and snow finches respectively increased fiber size and myofibril size compared to lowlanders (*p* values <0.05) (Fig 1C). Previous studies found two opposite phenotypes that lower or higher mitochondrial volume densities occurred in human or high-altitude deer mice [19–21]. We found that highland birds have higher total mitochondrial volume density and proportion of subsarcolemmal mitochondria in the pectoralis (*p* values <0.05) (Figs 1D and S2A). Overall, highland songbirds evolved substantial muscle phenotypes differing from these of lowland birds.

### High-altitude songbirds have improved insulin sensitivity

Skeletal muscle is the major tissue involved in the control of glucose homeostasis and a major determinant of resting energy expenditure [7, 22]. We observed that fasting plasma glucose was lower in highlanders compared to lowlanders, whereas plasma insulin was the same (Figs S2B and 1E). Furthermore, glucose and glycogen concentrations significantly increased in highland bird muscle (*p* values <0.05) (S2C Fig), highlighting a potential improvement in glucose utilization and insulin sensitivity. To further test this hypothesis, we compared the control of glucose homeostasis using glucose and insulin tolerance tests (GTT and ITT). Highlanders exhibited a more rapid normalization of blood glucose during GTT and also had a greater decrease in blood glucose concentration at 30 min and continued to 60 min after insulin injection (*p* values <0.05) (Fig 1F), indicating increased insulin sensitivity is an adaptive strategy in highlanders. To determine whether improved insulin sensitivity is associated with changes in energy expenditure, we conducted respirometry to measure metabolic features at rest. Highland songbirds showed a significant increase in respiratory exchange ratio (RER) (*p* values <0.05), but lower O_2_ consumption compared to lowlanders (ANCOVA with body mass as a covariate; F_2,17_ = 5.055, *p* value = 0.019) (Fig 1G and S1 and S2 Tables).

Additionally, discordant to the report that exercise improves glucose uptake through inducing expression of Glut4 and its translocation [23], lower relative activity was observed in highlanders compared to lowlanders (*p* values <0.05) (S2D Fig), indicating that songbirds keep quiet to reduce hypoxia stress and insulin sensitivity does not result from physical exercise at high altitudes. Taken together, improvement in insulin sensitivity is probably related with glucose utilization and muscle mass increase, as well as the decrease of oxygen consumption at the resting state.

### Molecular regulation of muscle physiology under hypoxia

**Fig 2:**
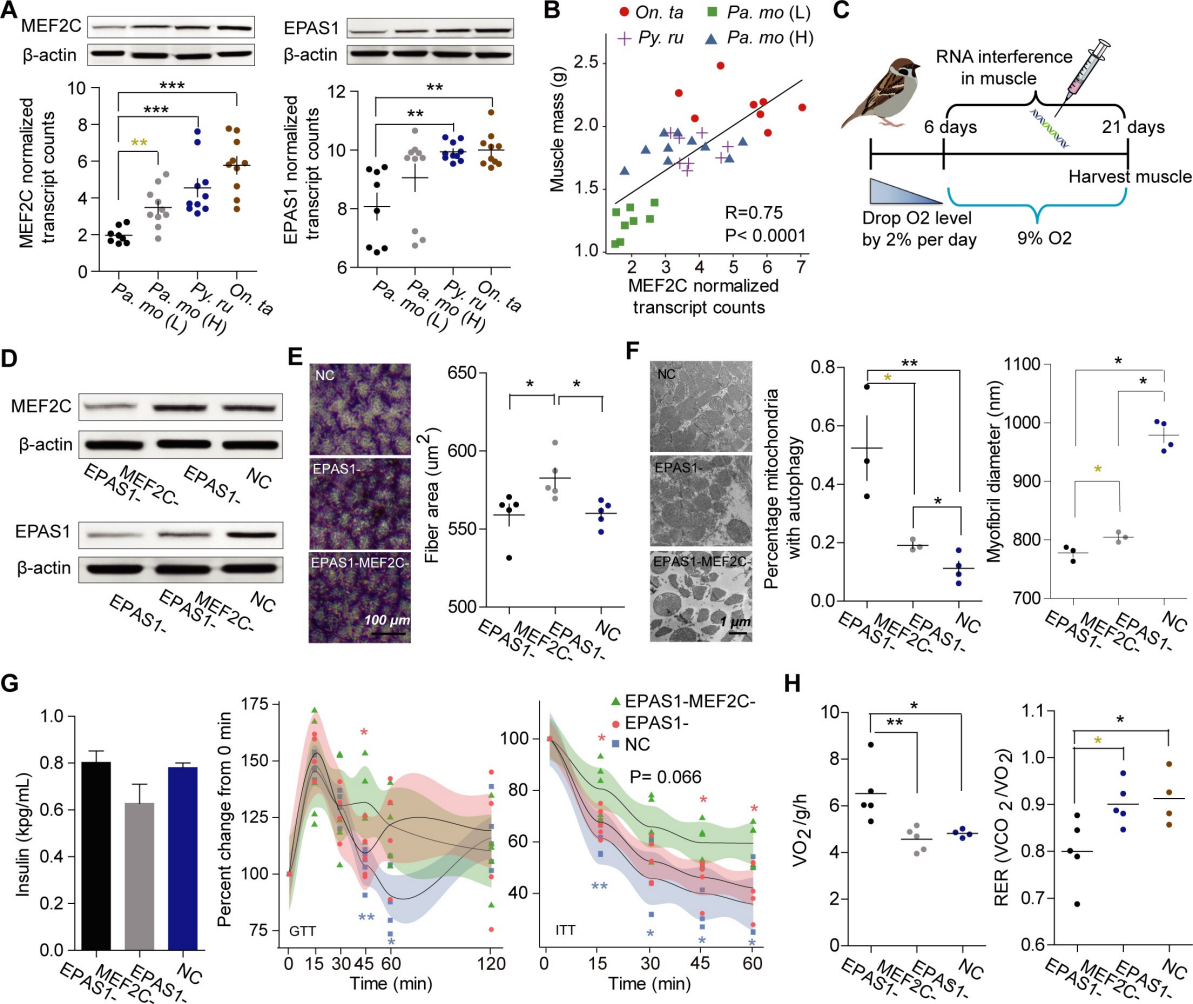
*MEF2C* and *EPAS1* maintain muscle phenotype and glucose homeostasis in chronic hypoxia. (A) The expression of *EPAS1* and *MEF2C* in the muscle was upregulated in snowfinches but only *MEF2C* was up-regulated in the highland tree sparrow [n = 8 for *Pa*. *mo* (L), and 10 for snow finches and *Pa*. *mo* (H)]. (B) The expression of *MEF2C* transcript was significantly correlated with muscle mass (n is the same with the above). (C) Schematic of gradual induction of chronic hypoxia protocol. Tree sparrows were exposed to gradual FiO_2_ reduction to 9%, injected with target siRNA every three days, and maintained for 15 days. (D) Protein levels of *EPAS1* and *MEF2C* in the muscle decreased after *EPAS1* and *MEF2C* knockdown. (E) SDH of muscle showed a reduction in fiber size in sparrows with downregulated *MEF2C* expression and high expression of *MEF2C* and *EPAS1*(n = 5 each). (F) TEM images of mitochondria showed severe mitophagy in birds with less *MEF2C* expression and knockdown of *EPAS1* improved protein breakdown (n = 3 for siRNA treatment, and 4 for control). (G) Glucose tolerance and insulin sensitivity were reduced in birds with *MEF2C* knockdown (n = 5 each). (H) knockdown of *MEF2C* increased oxygen consumption but reduced carbohydrate utilization (n = 5 for siRNA treatment, and 4 for the control group). NC is that birds were injected with equal normal saline.

To extract relevant information on the muscle phenotype, we then performed weighted gene correlation network analysis (WGCNA) to generate modules of genes correlated with muscle phenotypes using all expressed genes. Five, six and seven modules were identified to have a significant correlation with the first principal components of the PCA on the basis of 12 phenotypic traits at the *Pa*.*mo* (L)—*Pa*.*mo* (H), *Pa*.*mo* (L)—*Py*.*ru* and *Pa*.*mo* (L)—*On*.*ta* levels, respectively (correlation coefficient> 0.65, *p* values< 0.05) (S3A, S3B and S3C Fig). *MEF2C* was an up-regulated gene in all highland birds and identified as a shared hub gene in both the *Pa*.*mo*(L)—*Pa*.*mo* (H) and *Pa*.*mo* (L)—*Py*.*ru* networks of muscle phenotype-related modules (correlation coefficient> 0.65, *p* values< 0.05) (S3A and S3B Fig and S2 and S3 Datasets). We also found that *MEF2C* was a hub gene in yellow module of *Pa*.*mo* (L)—*On*.*ta* network, although correlation coefficient of this module was 0.50 (*p* value = 0.009) (S3C Fig and S3 Data). Genes of these modules containing *MEF2C* were related with muscle structure development (*p* value< 0.05), blood vessel development or remodeling (*p* values< 0.05) and insulin signaling pathway or negative regulation of insulin secretion (*p* values< 0.05) (S3A, S3B and S3C Fig). In addition, *EPAS1* played an important role in the *Pa*.*mo* (L)—*Py*.*ru* and *Pa*.*mo* (L)—*On*.*ta* turquoise modules and was also an up-regulated gene involving in hypoxic response (S3B and S3C Fig and S3 Data). The expression of *MEF2C* and *EPAS1* was significantly correlated with PC1 of muscle phenotypes (S3D Fig). Protein levels of these two genes in pectoral major muscle increased in snow finches; however, in highland tree sparrow, only *MEF2C* was up-regulated (Fig 2A). The expression of *MEF2C* showed a significantly positive association with muscle mass across species (R = 0.75, *p* value <0.0001) (Fig 2B). These results indicate that *MEF2C* and *EPAS1* could be crucial to maintain muscle mass and insulin response under hypoxia.

We next tested the function of *MEF2C* and *EPAS1* using RNA interference in the muscle when songbirds were exposed to low oxygen tension (9% O_2_). To avoid hypobaropathy resulting from a rapid drop in oxygen tension, we gradually decreased oxygen pressure by 2% per day from 20.9% to 9% and exposed siRNA-treated birds to 9% O_2_ for 15 days (Fig 2C). We found that all experimental birds underwent a reduction in body weight, but *MEF2C* and *EPAS1* double knockdown caused greater weight loss (S4A and S4B Fig), which resulted from a significant decrease in muscle mass (*p* values <0.05) (S4C Fig). This indicates that *MEF2C* rather than *EPAS1* maintains body weight through repression of muscle loss under chronic hypoxia. Protein expression levels of *MEF2C* and *EPAS1* markedly decreased after 15 days of hypoxia in the target siRNA groups (Fig 2D). Histological examination showed *EPAS1* knockdown exhibited significantly large muscle fibers (*p* values <0.05) (Fig 2E). A larger myofibril diameter occurred in control birds with both high expression of *MEF2C* and *EPAS1*(*p* values <0.05) (Fig 2F), which probably suppresses protein breakdown under hypoxia. In addition, *EPAS1* knockdown showed a lower capillary density (S4D Fig). We also observed severe mitophagy but no decrease of mitochondrial volume density when *MEF2C* and *EPAS1* were simultaneously interfered (*p* values <0.05) (Figs 2F and S4E). Overall, *MEF2C* and *EPAS1* can control muscle fiber or myofibril disintegration, capillarity, and mitophagy in hypoxia.

We further investigated whether *MEF2C* and *EPAS1* contributed to whole body glucose homeostasis. Birds with low *MEF2C* expression showed an insulin resistance with significantly higher blood glucose levels after 3 h of fasting and had similar insulin concentration. Additionally, these birds cleared glycemia more slowly in 45 min (also 60 min in hypoxia control group) during GTT and had a slow glucose clearance after 15 min during ITT than the other groups (*p* values <0.05) (Figs 2G and S4F). Furthermore, birds also exhibited reduced glycogen deposits and showed more relative activity but less RER at both resting and movement states (*p* values <0.05), which were observed with increasing oxygen consumption during the resting state (ANCOVA with body mass as a covariate; F_2,10_ = 7.158, *p* value = 0.012) (Figs 2H and S4G and S3 and S4 Tables). These results suggest that *MEF2C* might sustain insulin sensitivity, glucose homeostasis and low physical activity.

### Oxygen response in high- and low-altitude songbirds

**Fig 3:**
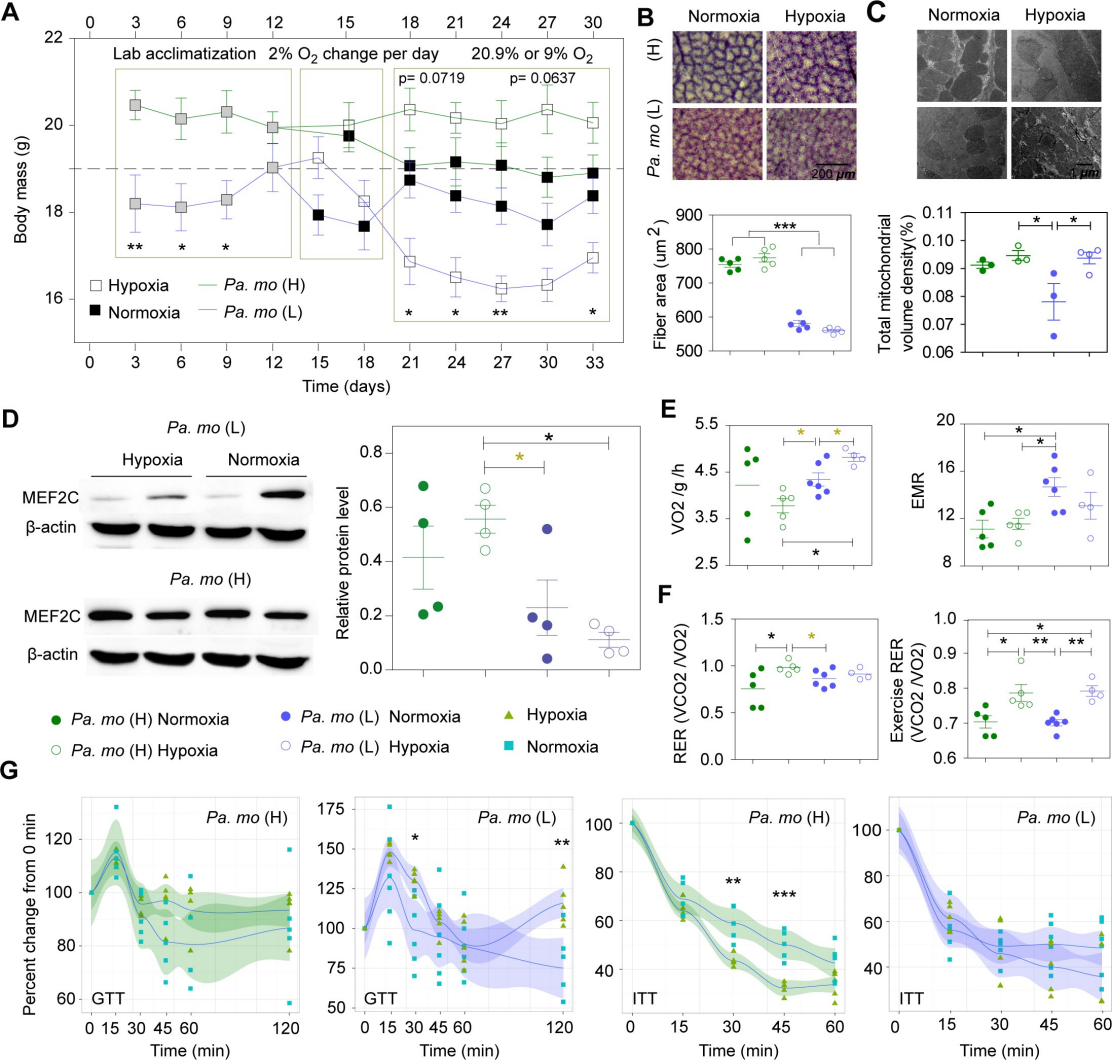
Muscle physiological plasticity and response to oxygen in high- and low-altitude tree sparrows. (A) Chronic hypoxia significantly reduced body weight of low-altitude tree sparrows but normoxia slightly decreased it in highlanders (n = 5 each). (B) Muscle mass was greater in high-altitude tree sparrows compared to lowlanders, which was not related to hypoxia(n = 5 each). (C) Mitochondrial volume densities were greater in high-altitude tree sparrow and hypoxia lowlanders (n = 3 each) compared to normoxia counterparts (n = 4). (D) High-altitude tree sparrow had a significant higher protein levels of MEF2C in hypoxia compared to hypoxia and normoxia lowlanders (n = 4 each). (E) Hypoxia induced high RMR in low-altitude tree sparrows (n = 6 and 4 for RMR) but not highlanders (n = 5 each). (F) Hypoxia increased resting RER only in highlanders but exercise RER both in high- and low-altitude tree sparrows. (G) High-altitude tree sparrow improved insulin sensitivity in hypoxia compared to normoxia (n = 5 each). Hypoxia was unable to change insulin sensitivity but induced glucose intolerance and increased insulin contents in low-altitude tree sparrows (n = 6 and 5 for normoxia and hypoxia).

To exclude the effects of plasticity and temperature on the high-altitude songbirds, we tried to perform hypoxia (9%) and normoxia (20.9%) treatments at 25°C for highland tree sparrows and rufous-necked snow finch (natives from 4200m and 4400m, respectively). The deaths usually occurred during the first week for all wild-captured songbirds but snow finches also had a second outbreak of death during the last week because of the persistent losses of body weight (S5A and S5B Fig). High-altitude tree sparrows were slightly decreased body weights and muscle masses in normoxia but no significant difference compared to hypoxia treatment (Figs 3A and S5C). Flight muscle mass, muscle fiber size and capillary per fiber of high-altitude tree sparrows were increased in normoxia and hypoxia compared to low-altitude counterparts (*p* values < 0.05; Figs 3B, S5C and S5D) despite body weight in normoxia closed to these of low-altitude tree sparrows (S5A Fig), showing that these were probably not plastic in adult high-altitude tree sparrows. Mitochondrial volume density of high-altitude tree sparrows was similar to this of lowlanders in normoxia, suggesting a plasticity (Fig 3C), but high-altitude tree sparrows in hypoxia did not have an obvious mitophagy compared to in normoxia and lowland tree sparrow (S5E Fig). Additionally, hypoxia slightly induced but did not decrease the protein level of *MEF2C* in high-altitude tree sparrows which was still increased *MEF2C* abundance compared to lowlanders both in hypoxia and normoxia(*p* values <0.05, Fig 3D). High-altitude birds showed lower glycemia after three hours fasting (*p* values <0.05; S5F Fig), a slight lower resting oxygen consumption (Fig 3E) and a significant higher RER and exercise RER in hypoxia compared to in normoxia (p values <0.05; Fig 3F). Highland tree sparrow in hypoxia but not normoxia decreased oxygen consumption compared to lowland tree sparrows both in normoxia and hypoxia (ANCOVA with body mass as a covariate; F_2,11_ = 9.958, *p* value = 0.003) (Fig 3E and S3 and S5 Tables). We also found that insulin sensitivity was increased in 30 min and 45 min (Fig 3G) but similar insulin level was in hypoxia compared to normoxia (S5G Fig), which likely drove glucose utilization and glycogen storage in high-altitude tree sparrows (*p* value <0.01; S5H Fig). Overall, hypoxia failed to induce muscle loss in highland adult tree sparrows but improved insulin sensitivity to sustain glucose metabolism.

To examine the effect of hypoxia on the lowland tree sparrows, we compared the changes in muscle physiology of lowland tree sparrows under hypoxic and normoxic conditions. Body weight significantly decreased on the third day and this low body weight was sustained until the 15th day under 9% oxygen conditions (*p* values <0.05) (Fig 3A). Hypoxia induced a significant decrease in muscle mass (S5C Fig). Hypoxic exposure causes mitochondrial autophagy at the cellular level [24]. Surprisingly, we observed an increase in mitochondrial volume density although there was an obvious mitophagy in hypoxic birds (*p* values <0.05) (Fig 3C), which was probably caused by an increase in metabolic rate (ANCOVA with body mass as a covariate; F_1,7_ = 5.740, *p* value = 0.048) (Fig 3E and S3 and S6 Tables). Hypoxic birds had lower glycemia after three hours fasting (S5F Fig) and higher RER of exercise, suggesting the increase of glucose utilization (*p* values <0.05) (Fig 3F). However, we found glucose intolerance during GTT but failed to find the change on insulin sensitivity during ITT in hypoxia birds (Fig 3G). High insulin content in hypoxia probably contributed to the increase of glucose utilization and glycogen storage (*p* values <0.05) (S5G and S5H Fig). From our experiment, hypoxia slightly decreased the protein abundance of *MEF2C* (Fig 3D). These results suggest that lowland native exposure to chronic hypoxia undergo muscle loss and increases energy expenditure rather than the similar muscle physiology of highlander, indicating a different genetic background between high- and low-altitude populations.

### Genetic basis of muscle physiology in high-altitude songbirds

**Fig 4:**
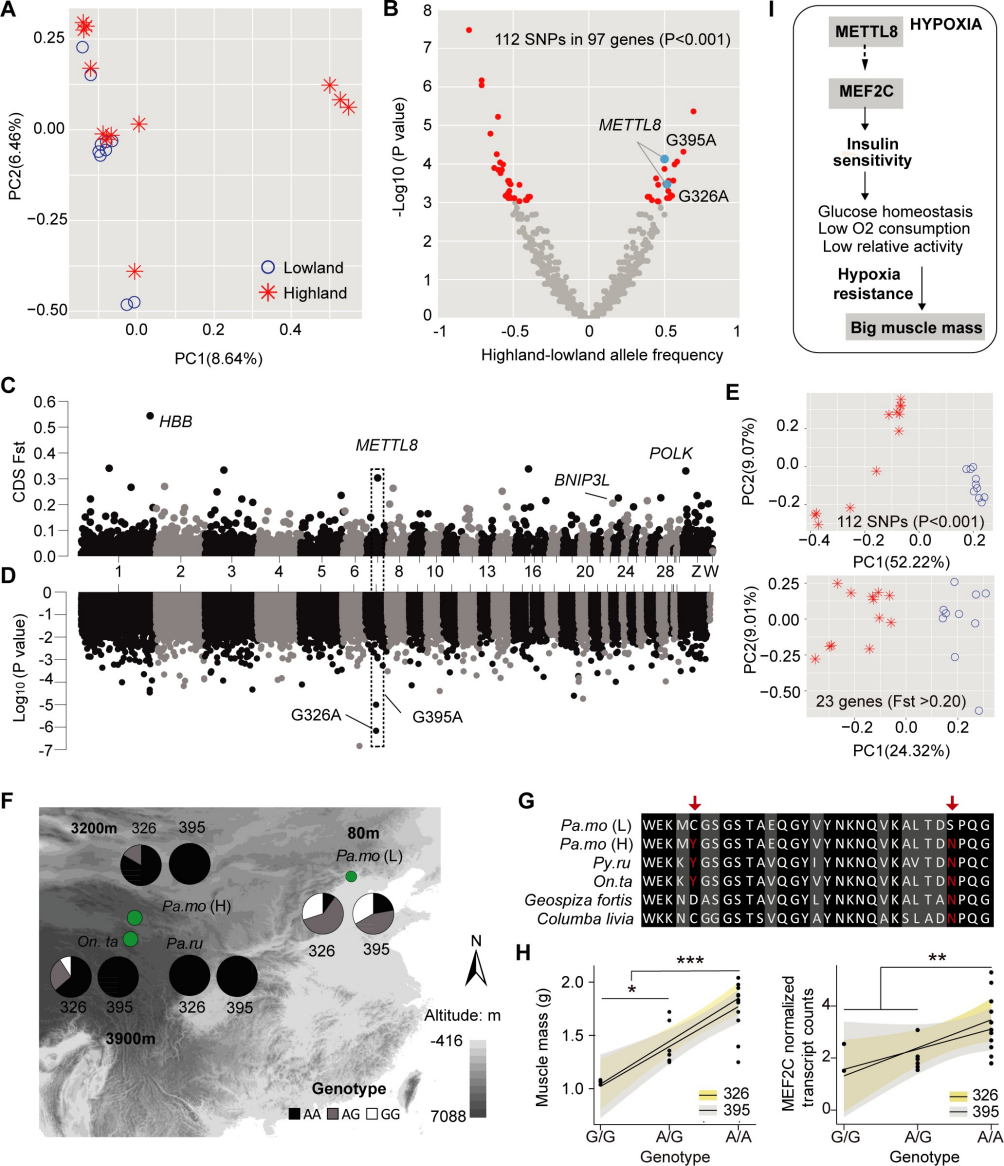
Genetic variation in the *METTL8* gene. (A) Principle component analysis of the genetic data (SNPs) for individuals from altitudinal populations. (B) A volcano plot showing allele frequency differences between highland and lowland tree sparrow RNA-seq samples on the x-axis and the–log_10_ P values on the y-axis. The 112 significant SNPs in 97 genes were labeled in red (n = 10 and 12 for highland and lowland populations). (C) Manhattan plot: Outcome fixation index (F*st*) for potential CDS region. (D) Genome-wide association analysis between muscle mass and the genotypes at nearly 120000 SNPs. (E) Principle component analysis of the SNPs of 112 SNPs (P <0.001) and 23 genes with high F*st*. (F) Genotypes of the lowlander and highlanders at two *METTL8* SNPs. The putatively advantageous homozygote is shown in black, heterozygote was shown in gray, and the non-advantageous homozygote is indicated in white. (G) Amino acid alignment of *METTL8* across five species. Putatively advantageous mutations are shown in red. (H) *METTL8-A* alleles at site 326 and 395 are associated with greater muscle mass (R = 0.88 and 0.83, respectively, n = 19) and *MEF2C* expression (R = 0.66 and 0.48, respectively, n = 18) in tree sparrow altitudinal populations. (I) Simplified schematic of hypoxia resistance mechanism in high altitude songbirds. Convergent mutations in *METTL8* can activate *MEF2C* transcription, leading to the upregulated expression of *SLC2A12* that further promotes insulin sensitivity, and accompanied by *EPAS1*-controlled muscle development and maintenance.

Next, we identified genes with high amounts of genetic differentiation between altitudinal tree sparrows. We performed a principal component (PC) analysis that suggested that individuals from the two populations could not be distinguished on PC1 of CDS region SNPs, with obvious overlap between high- and low-altitude populations (Fig 4A). Moreover, the weighted *Fst* between the two populations was 0.014 (mean *Fst* = 0.008), indicating that the overall genetic differentiation was extremely low. In the RNA-seq data, we identified 112 SNPs in 97 genes with significantly different allele frequencies (*p* value <0.001), and 23 genes with high fixation index (CDS region *Fst* >0.20) between high and low altitudinal populations (Fig 4B and 4C and Table 1). PC1 of a principle component analysis of SNPs in these candidate genes explained over 20% of the observed variations in the data and could distinguish the two altitudinal populations (Fig 4E).

**Table 1:** Candidate genes with nonsynonymous substitutions from high CDS differentiation between altitudinal populations (*Fst* >0.20). Four genes with high genetic differentiation are identified and three of these have convergent substitution in two or three high-altitude songbirds.

Gene	Fst	Mutant (LA *vs*. HA)	△AF	Fisher P-Value	AAS	Convergence	P-Value (plink)	P-Value (lme4)
*HBB*	0.54	A238G	0.71	6.75E-7	Ser80Gly	*Py*. *ru*	4.60e-5	0.54
		G334A	0.79	3.34E-8	Val112Ile	*Py*. *ru*	3.49e-5	0.67
*POLK*	0.33	C2426A	0.38	2.08E-3	Thr809Asn	Unique	0.0033	0.38
		A2429G	0.38	2.08E-3	Tyr810Cys	Unique	0.0033	0.38
*METTL8*	0.30	G326A	0.52	3.36E-4	Cys109Tyr	*Py*. *ru*, *On*. *ta*	6.93e-7	2.71e-5
		G395A	0.50	7.45E-5	Ser132Asn	*Py*. *ru*, *On*. *ta*	9.99e-6	0.01
*BNIP3L*	0.21	G118A	0.30	5.49E-3	Ser40Gly	*Py*. *ru*, *On*. *ta*	0.0422	0.51
		A124G	0.25	1.43E-2	Gly42Ser	*Py*. *ru*	0.1531	0.25

LA, low-altitude allele; HA, high-altitude allele; △AF, the difference of allele frequency between altitudinal populations; AAM, amino acid substitution.

Remarkably, among these 23 candidates were four genes with highly differentiated SNPs and with nonsynonymous changes which of three genes were convergent with high-altitude endemic species and were good candidates for influencing hypoxia response (Table 1). One of these genes was hemoglobin subunit beta (*HBB*), of which two substitutions (Ser80Gly and Val112Ile) were convergent with the *Py*. *ru*. The Ser80Gly mutant in Andean high-altitude house wren (*Troglodytes aedon*) and Val112Ile mutant in band-winged nightjar (*Hydropsalis longirostris*) are thought to increase Hb–O_2_ affinity (S6A and S6B Fig) [25, 26]. One candidate gene was methyltransferase-like 8 (*METTL8*), which has been associated with metabolic disease in humans [27], body weight in chicken [28] and myogenic differentiation by influencing myogenic gene expression (*SRF* and *MEF2*) in mouse [29]. The other candidate gene, BCL2 interacting protein 3 like (*BNIP3L*) which was crucial for hypoxia-induced mitophagy and tumor progression [30–32], had two convergent missense mutations with the *Py*. *ru*. but only one with *On*. *ta*.

We used an association test to investigate whether any of the SNPs with the most significant allele frequency differences between the populations are associated with body and muscle mass. Notably, two SNPs in the *METTL8* gene encoding two missense mutations occurred in all high-altitude birds and were significantly related with muscle mass and body weight (Figs 4D, S6C–S6F and Table 1). The *METTL8* gene showed a G-to-A transition at site 326 and 395, causing cysteine-to- tyrosine and serine-to- asparagine nonsynonymous substitutions, respectively, with a higher A frequency in high-altitude songbirds (Fig 4G and 4F). Homozygous individuals for the highland genotype had greater body weight and muscle mass than both heterozygous and homozygous individuals for lowland genotype (*p* values <0.05) (Figs 4H and S6G). *MEF2C* expression was also markedly upregulated in the homozygous highland genotype (*p* values <0.05) (Fig 4G). These results suggest that the convergence in *METTL8* may contribute to insulin sensitivity and further muscle mass increase via activating *MEF2C* in high-altitude birds (Fig 4I).

## Discussion

In this study, we revealed convergent physiological and genetic mechanisms of the skeletal muscle resistance to hypoxia in high-altitude songbirds. High-altitude birds suffer from severely limited oxygen supplement due to decrease in the partial pressure of oxygen. Therefore, the physiological responses to highland hypoxia are generally found by altering oxygen utilization in pectoralis muscle [9]. We found an increase of muscle mass resulting from improvement in insulin sensitivity in high-altitude songbirds. Muscle mass and phenotype variations in highland songbirds are probably associated with the flight performance in hypobaric environment and thermogenesis in cold temperature [17, 33]. Animals usually increase their resistance to environmental challenges by altering their behavior responses, such as torpor and hibernation in birds and rodents in response to cold stress [34]. Therefore, reduced physical activity can decrease energy expenditure in high-altitude hypoxia. Additionally, it is confirmed an increase in carbohydrate consumption and a lower plasma glucose in high-altitude animals [10, 35], whilst how birds regulate blood glucose remains unclear. Here we present the first compelling evidence that improving insulin sensitivity may play an important role on carbohydrate utilization and low physical activity in high-altitude songbirds.

Comparative genome and transcriptome analyses across altitudinal species have revealed that convergent evolutionary changes in expression and sequence levels are mainly related to energy metabolism [12, 14]. However, only a few studies have confirmed that these convergent signatures do indeed improve individual fitness to high-altitude environments and physiological evidence are currently unavailable. In this study, combined transcriptomic and physiological analyses demonstrated a convergence on muscle physiology and energy metabolism in high-altitude songbirds. In addition, we verified using RNA interference that convergent *MEF2C* signaling was pivotal to maintain muscle mass and glucose homeostasis and thus decreased physical activity under hypoxia. However, *EPAS1* controls mitophagy, protein breakdown and capillarity, which suggests the evolutionary conservation of function in birds and mammals [36, 37]. *MEF2C* is a key transcriptional regulator that drives skeletal muscle development during embryogenesis, and hippocampal-dependent memory [38, 39]. Our findings are discordant with these observations, as we highlight a novel function for *MEF2C* in sustaining postnatal muscle mass and glucose metabolism under hypoxia in songbirds.

Our previous study revealed that high-altitude tree sparrows colonized the QTP for millennia and underwent weak genomic differentiation with the lowland population, whereas polygenic adaptation rather than phenotypic plasticity promoted muscle phenotypic adaptation to high-elevations [26]. Consistent with this result, low-altitude tree sparrows suffered from muscle loss and increased energy expenditures under hypoxia, which was conserved in many mammals and even human [3–5], but converse with high-altitude songbirds. In this study, we identified multiple candidate genes for muscle physiological adaptation to hypoxia in highland tree sparrows including *HBB* which was the first most highly differentiated gene between the populations and might increase Hb-O_2_ affinity at two nonsynonymous substitutions [25, 26]. In addition, lowland birds had an increase in capacity of mitochondrial autophagy and muscle loss under chronic hypoxia, suggesting that convergent sites in *BNIP3L* of highland songbirds probably prevented mitophagy and apoptosis under hypoxia as reported by previous studies [30–32]. We further investigated one of the candidate genes, *METTL8*, in detail. Two missense mutations in *METTL8* with high genetic differentiation between altitudinal populations was significantly associated with body weight, muscle mass and *MEF2C* expression. These two mutations in high-altitude tree sparrows also occurred in other two endemic species of the QTP, suggesting that convergent substitutions of this gene may come from standing variations in high-altitude songbirds. *METTL8* is identified as a new mRNA m3C writer enzyme, and plays a crucial role in myogenic differentiation and metabolic disease [27, 29]. Knockdown of *METTL8* decreased *MEF2* expression in embryonic mesenchymal cells [29]. These results highlight that high-altitude genotype of *METTL8* could cause unique muscle physiological resistance to hypoxia by regulating *MEF2C* transcript abundance, and rapid evolution of muscle hypoxia tolerance can be explained by large-effect alleles rather than several loci of small effect.

## Methods

### Ethics statement

All procedures performed on animal were approved by the Animal Care Committee of the Institute of Zoology, Chinese Academy of Sciences. The approval number was IOZ20160049.

### Data collection and sampling

We measured 467 museum specimens belonging to 3 Passeridae species from the National Zoological Museum, Institute of Zoology, Chinese Academy of Sciences (see S1 Data). Using mist-net in summer of 2016, highland birds were caught from Qinghai Province at about 3,200 m (10 tree sparrows) and 3,900 m (10 rufous-necked snow finch and 10 white-rumped snowfinch), and lowland birds (n = 8) of the tree sparrow were from Yanqi Lake of Beijing at 80 m. Blood samples were collected and centrifuged at 2,500 rpm for 20 min after fasting for two hours. Pectoral major muscle was dissected immediately following euthanasia. Plasma and tissues were flash-frozen in liquid nitrogen and stored at -80°C until analysis.

### Behavioral measures

In the field, we mainly conducted direct observation on birds and combined video record on captive birds. Briefly, subjects were observed continuously for a period of 2 h and behaviors were recorded. In the laboratory, general activity patterns were measured using video recordings, and each treatment was recoded for at least four hours in daylight. The behaviors were divided into three parts, namely, eating, moving and sedentary. Then we calculated the time when birds had been actively eating, moving and inactive (sedentary).

### Histology and transmission electron microscopy

Oxidative muscle type and capillarity were analyzed as previously described [9]. Briefly, the pectoral major muscle was dissected and samples were taken third way along the sternum, covered with OCT, and frozen in isopentane (cooled in liquid N2). Muscle sections (10 microns) were obtained in a Cryostat Microtome (Leica CM900, Germany) at -20°C. Succinate dehydrogenase and alkaline phosphatase staining were performed to identify oxidative muscle fibers and muscle capillaries, respectively. The sections were imaged using 10X objective of light microscopy (Leicas DM750) and at least eight images were analyzed for each sample using image J software.

Flight muscles were removed from an intermediate depth and then fixed at 4°C for 24–48 h in 2% glutaraldehyde in 0.1M PBS buffer at pH 7.4. Small muscle blocks (2 mm × 2 mm) were prepared and post-fixed in 1% osmium tetroxide in 0.1 M PBS for 1 h, dehydrated in a graded ethanol series (50%, 70%, 70%, 95%, 95%, 100%, 100%), and embedded in epoxy resin. Ultra-thin sections were cut on a Leica UC7 ultramicrotome and placed on copper grids. The sections were post-stained with uranyl acetate and lead citrate. Images were collected using a transmission electron microscope (Tecnai G2 F20 TWIN TMP, USA). We measured mitochondrial volume density using stereological methods as previously described [40, 41]. Grid size of 90 nm was used and mitochondrial volume was estimated through number of intersection in a mitochondrion dividing total number of intersection at a square size of 4,460 × 4,460 nm. Fracted mitochondria were considered to be autophagy and the ratio of number of fracted mitochondria to normal mitochondria were calculated. We identified mitochondria nearby myolemma and not surrounded by myofibril as subsarcolemmal mitochondria.

### Plasma insulin and tissue glycogen measurement

Fasting plasma was obtained from each species. Insulin concentrations were detected using insulin ELISA kit (Cloud-Clone), and muscle glycogen were determined with a relative assay kit (Solarbio) according to the manufacturer’s instructions.

### Glucose quantification

Flight muscle (n = 10 each) and plasma (n = 7 each) metabolites were extracted by methanol/chloroform protocol, as previously described [42]. Approximately 1 mL of a chloroform/methanol/distilled water mixture (1:4:1) was added to Eppendorf tubes containing ∼50 mg flight muscle powdered in lipid nitrogen. Then, 450 μL of methanol/distilled water mixture (8:1) was added to Eppendorf tubes containing ∼50 μL plasma. 20 μg, and 10 μg of heptadecanoic acid as well as decanoic acid as double internal standard were added into the muscle and plasma tube, separately. After vortexing for 10 s, the samples were placed on ice for 15 min and then in a sonication bath for 15 min. After centrifugation at 12,000 rpm for 15 min at 4°C, 200 μL of the supernatant were transferred to a 2 mL auto-sampler vial and dried in a vacuum oven. Dried samples were derivatized using methoxyamine hydrochloride solution (50 μL of 15 mg·mL^−1^ methoxyamine in pyridine). The mixture was kept for 16 h at room temperature for methoxymation, and then 50 μL of TMSFA containing 1% TMCS were added for trimethylsilylated. After 1 h trimethysilylation, 30 μL of hexane was added and then transferred to an insert in a 2-mL autosampler vial for GC-MS analysis. A 2 μL of the derivatized sample was injected by an Agilent 4683B GC auto-sampler (Agilent Technologies, Atlanta, GA, USA) into an Agilent 6890N gas chromatograph with 5973 mass spectrometry at 250°C without splitting. The gas chromatography-mass spectrometry analysis program was as follows: the GC oven temperature was set at 90°C for 1 min, and then increased to 175°C at 5°C min^−1^, held for 3 min, increased to 270°C at 3°C min^−1^, 310°C at 20°C min^−1^, and finally held for 15 min. Identification of glucose from GC-MS analysis was performed by using glucose (Sigma-Aldrich) as standard.

### Respirometry

We measured the rates of oxygen consumption VO_2_ by open flow-through respirometry (Foxbox). For resting metabolic rate (RMR), birds were maintained in a 1.0L transparent metabolic chamber with an air flow rate of 500 mL/minute and O_2_ and CO_2_ concentration within the chamber were recorded for over 15 min. The lowest 1-min average of oxygen consumption was taken as the RMR. For exercise oxygen consumption, we used a metabolic flight wheel as previously described [43]. An incurrent flow rate of 5 L/min and a subsampling rate of 100 mL/min were used. We exercised the birds using an initial speed of wheel rotation (approximately 0.3 m/s) and increased the rotation speed by approximately 0.1 m/s every 2 min until birds could no longer keep position and VO_2_ no longer increased with increasing speed. We took the mean of two minutes of peak O_2_ consumption and CO_2_ production to calculate exercise RER. The tests lasted for 20 min. We corrected for the effects of body mass on RMR and exercise oxygen consumption using an analysis of covariance (ANCOVA) with body mass as covariate. Statistical tests were performed using IBM SPSS 22 (SPSS Inc.) with a significance level set to *p* value = 0.05.

### RNA sequencing and differential expression analysis

Total RNA was extracted from each of the 38 sample (8 lowland tree sparrows, 10 highland tree sparrows, 10 rufous-necked snow finches, and 10 white-rumped snow finches) using Trizol RNA isolation reagents (Invitrogen Corp., Carlsbad, CA). RNA integrity was assessed using the RNA Nano 6000 Assay Kit of the Agilent Bioanalyzer 2100 system (Agilent Technologies, CA, USA). A total amount of 3 μg RNA per sample was used as input material for the RNA sample preparations. Sequencing libraries were generated using NEBNext Ultra RNA Library Prep Kit for Illumina(NEB, USA) following manufacturer’s recommendations and index codes were added to attribute sequences to each sample.

Trimmomatic [44] was used to filter reads containing adapter, reads containing ploy-N and low quality reads based on read quality checked with FASTQC [45]. The parameters used were as follows: sliding window = 4-bp; Phred33 quality scores = 20; min read length = 50. Adapter sequences, when detected, were removed. Clean data with high quality were mapped from each species to respective genomes [16] using STAR with default parameters [46]. We used the reciprocal best-hit method to generate tree sparrow-rufous-necked snowfinch and tree sparrow- white-rumped snowfinch orthologs, respectively. The orthologs shared by three species were obtained by intersecting the lists of the above two orthologs. After the reads were mapped to the reference genomes, expression quantification of the genes and transcripts were performed using RSEM [47]. Expression levels for genes with one-to-one orthologs in all three bird species (n = 12,951) were normalized with a RLE (relative log expression) method across muscle samples [48, 49].

To identify genes related with flight muscle variation across altitudinal songbirds, we performed differential expression analysis. Differentially expressed genes (DEGs) were calculated based on the negative binomial distribution and independent filtering was enabled in the program Deseq2 with a false discovery rate (FDR) < 0.05 based on Benjamini-Hochberg method to account for type I error in identifying significantly DEGs [50]. The cut off values for log_2_-fold change were set at 0.59 and -0.59. Only genes with count number > 10 in at least four samples were included in differential gene expression analysis (see S2 Data).

### Weighted gene co-expression network analysis (WGCNA)

We performed WGCNA to identify gene modules associated with muscle phenotypic variance and its potentially genetic basis. The first three principal components of the PCA on 12 phenotypes were used to analysis involved in body weight, lipid droplet volume density, muscle fiber (fiber density, fiber area and myofibril size), capillarity (capillary density, capillary size and capillary per fiber) and mitochondrial phenotypes (distribution of subsarcolemmal mitochondria, volume densities of subsarcolemmal and intermyofibrillar mitochondria, and total mitochondria volume density). All expressed genes were analyzed. Briefly, we constructed the weighted gene co-expression network using the normalized, log_2_-transformed counts to analyze all expressed genes with the blockwiseModules function in WGCNA [51] for the *Pa*.*mo* (L)—*Pa*.*mo* (H), *Pa*.*mo* (L)—*Py*.*ru* and *Pa*.*mo* (L)—*On*.*ta* levels, respectively. For our analysis, the parameters used were as follows: for *Pa*.*mo* (L)—*Pa*.*mo* (H) network, maximum block size = 5000 genes, power (β) = 4; for *Pa*.*mo* (L)—*Py*.*ru* network, maximum block size = 5000 genes, power (β) = 12; for *Pa*.*mo* (L)—*On*.*ta* network, maximum block size = 5000 genes, power (β) = 18; minimum module size = 25; minimum height for merging modules = 0.25; maximum height for cutting the tree = 0.90. The remaining parameters were kept at the default settings.

Co-expression modules associated with phenotypes were identified using a principal component analysis (PCA) of gene expression with the blockwiseModules function in WGCNA. Each module was summarized by an eigengene, which is the first principal component of the scaled module expression. Trait-related modules were decided by correlation coefficient> 0.65 and *p* values< 0.05. To specify genes potentially explaining muscle phenotypic variation, we identified hub genes which may be central to the architecture of the regulatory networks represented by each co-expression module. The hub genes were calculated by their first principal component (PC1), the module eigengene (a summary of overall module expression) [52]. Genes which had higher degree (top 25% of genes in module) were identified as hub genes in trait-related module network. We then implemented gene ontology categories within modules positively and negatively correlated with muscle traits using G:PROFILER (FDR < 0.05) [53]. According to the results of functional GO enrichment analysis, intramodular hub genes were identified as candidate genes potentially driving muscle variation.

### Genetic variation analysis

Clean data of RNA sequence from high and low altitude populations (12 *vs*. 10 individuals) was aligned to tree sparrow CDS reference using BWA [54] with default parameters. Only reads that were mapped uniquely to the reference were retained. After high-quality filtering with custom scripts, SNPs were called on all 22 individuals with Samtools. Local realignment over indel positions were performed using GATK [55]. SNPs with missing data in two or more individuals in altitudinal populations were excluded. Allele frequencies and fixed index (*Fst*) were estimated using Vcftools [56]. Fisher’s exact test was used to determine the statistical significance of the allele frequency differences between highland and lowland populations at exonic SNP positions. Genotype–phenotype correlations between about 120000 SNPs and muscle mass as well as body weight was performed using Plink though the default settings. We further verified the association from results of Plink using a linear mixed model as implemented in the R package lme4 [57]. Candidate genes and SNPs were identified according to *Fst* >0.3, Fisher’s exact test P-value <0.001 and P-value transformation of genotype–phenotype association >4.

### Western blot

Western blots were performed on samples of pectoral major muscle, as previously described [58]. 50 mg of muscle were grinded with liquid nitrogen and then the powder was lysed in 1 ml lysis buffer, 50 mM Tris HCl (pH 7.5), 150 mM NaCl, 5 mM EDTA, 0.5% Nonidet P-40, and completeMini protease inhibitor cocktail (BC3640; Solarbio). The samples were placed on ice for 4 min and then centrifugated at 12,000 rpm for 15 min at 4°C. 350 μL of the supernatant were transferred to a 2 mL auto-sampler vial and were diluted by 3 fold of loading buffer. Protein concentration was quantified using the BCA Protein Assay Kit (PC0020; Solarbio). Supernatant were then separated using SDS-PAGE for 1 h at 140V and transferred to PVDF for 2 h at 90V. Nonspecific antibody bind in was blocked in 5% nonfat dry milk powder in 1 Tris-buffered saline for 1 h. Blots were incubated in 1% nonfat dry milk powder in Tris-buffered saline with 0.05% Tween 20 overnight at 4 C with the following antibodies: rabbit anti-MEF2C (bs-4130R; 1:1,000; Bioss), rabbit anti-EPAS1 (bs-1447R; 1:1,000; Bioss), and mouse anti-β-actin (bsm-33036M; 1:1,000; Bioss). Blots were incubated with goat anti-rabbit (bs-0295G; Bioss) or goat anti-mouse antibodies (bs-0296G; 1:3,000; Bioss) for 1 h at room temperature. Blots were detected using protein detection reagents (AY0371; Solarbio). Membranes were quantified using the ImageJ software and expression was quantified.

### *In vivo* experiments and RNA interference

The experimental birds were transported to the laboratory at the Institute of Zoology, Chinese Academy of Sciences after capture. Birds were weighed and housed individually in screen cages to avoid bird injury (35 cm × 35 cm × 35 cm) at a constant temperature (25±1°C) and 12 h:12 h L:D photoperiod. After acclimation to laboratory for about 14 days, 15 individuals with similar weights were randomly separated into three groups for hypoxia experiment. For hypoxia trails, a hypoxic chamber (FLYDWC-50; Fenglei Co., Ltd, China) was used to control oxygen concentration in normobaric state (20.9kPa). After gradual decrease of oxygen concentration from normoxia (20.9%) to hypoxia (9%), two groups of birds (n = 5 each) were injected with 1 nmol of a mixed double-stranded *EPAS1* siRNA or *MEF2C*+*EPAS1* siRNAs (si*MEF2C*: CCAACAAACTGTTCCAGTA; GCCGAACAAATTCAGATAT; GCACTGATATGGACAAAGT; si*EPAS1*: TCATCCATGTGACCATGAA; GCATGAGTACCATCTTCCA; TGATCTACAATACCCGAAA; Ribobio, Guangzhou, China), respectively. And then these three groups were maintained for 15 days in 9% oxygen tension. Normoxia and hypoxia control groups were injected with equal normal saline. siRNA was vertically injected to 2 millimetres deep of the pectoralis major using microsyringe. The site of injection was closed to bird carina about 0.5 cm. Birds were injected with target siRNA and weighed every three days during cage cleaning for a brief period (<1 h) and were provided with water and mixed seed *ad libitum*.

High-altitude tree sparrows and rufous-necked snow finch were respectively captured from 4200m and 4400m, and then transplanted to transported to the laboratory at Tibet Agriculture & Animal Husbandry University in Linzhi (elevation 3000m). Birds were kept at a constant temperature (25±1°C) and 12 h:12 h L:D photoperiod for 12 days. After acclimation, 12 tree sparrows and 7 rufous-necked snow finches with similar weights were randomly separated into two groups for hypoxia and normoxia experiment. Oxygen concentration were gradually increased and decreased from 15% to 20.9% and from 15% to 9% (2% per day). And then birds were maintained for 15 days in 20.9% and 9% oxygen tensions.

### Glucose and insulin tolerance tests

For the GTT, an oral gavage of 50% D-glucose (3 mg/g body wt; Sigma) was performed after 16 h fasting. Blood glucose was measured at 0, 15, 30, 45, 60, and 120 min via the medial metatarsal vein. For the ITT, human insulin (1.5 mU/g body wt; Novonordisk) was injected to 2 millimetres deep of the enterocoelia using microsyringe after the birds underwent three hours of fasting. Blood glucose was measured at 0, 15, 30, 45, and 60 min via the medial metatarsal vein. Glucose and insulin were measured using an Accu-Check blood glucose meter (Roche Diagnostics) and an insulin ELISA kit (Cloud-Clone), respectively.

### Statistical analyses

One-way ANOVA to analyse was used to determine statistical significance across species and treatments. But we also performed two-tailed Student’s t-test if no statistical significance of comparative analyses using above method. For all figures, *p* value < 0.05 were considered significant. The black and green asterisks respectively present significant differences for One-way ANOVA and two-tailed Student’s t-test. *P < 0.05, **P < 0.01, ***P < 0.001.

## Supporting information

S1 Fig Altitudinal distribution and body variation. The relationship between log10 body weight (g) mass and elevation (meters) in three songbirds.
(TIF)

S2 Fig Muscle phenotypes and metabolic variation. (A) TEM images of mitochondria showed an increase in proportion of subsarcolemmal mitochondria but no mitophagy in high-altitude birds (n = 6 each). (B) Fasting plasma glucose was lower in highlanders compared to lowlanders (n = 7 each). Relative content is the ratio of the peak area of metabolic intermediate to the peak area of the internal standard (heptadecanoic acid). (C) Relative levels of glucose and glycogen contents were increased in highlanders (n = 10 each). (D) Physical activity levels in high and low altitude birds (n = 8 for lowland tree sparrow and rufous-necked snowfinch, and 6 for highland tree sparrow).
(TIF)

S3 Fig Weighted gene correlation network analysis (WGCNA) based on all expressed genes. (A) Five modules were identified in *Pa*.*mo* (L)—*Pa*.*mo* (H) level using WGCNA analysis. *MEF2C* was identified as a hub gene in yellow module. (B) Six modules were identified in *Pa*.*mo* (L)—*Py*. *ru* level using WGCNA analysis. *MEF2C* and *EPAS1* were identified as the hub genes in magenta module and turquoise module, respectively. (C) Seven modules were identified in *Pa*.*mo* (L)—*Ta*. *on* level using WGCNA analysis. *EPAS1* were identified as a hub gene in turquoise module. *MEF2C* was also a hub genes in yellow module, although the correlation coefficient was 0.50 (*p* value < 0.01). (D) *MEF2C* and *EPAS1* were significant correlated with PC1 of muscle phenotype.
(TIF)

S4 Fig *MEF2C* and *EPAS1* effect glucose homeostasis and muscle phenotypes. (A) Body weight variation of different siRNA treatments under chronic hypoxia (n = 5 each). (B) Birds with less *MEF2C* expression showed a dramatic decrease in body weight and had significant increase in loss of body mass than other group (n = 5 each). (C) The loss of muscle mass contributed to reduction of body weight in knockdown of *MEF2C* (n = 5 each). (D) knockdown of *EPAS1* prevented angiogensis in chronic hypoxia (n = 5 each). (E) No difference in total mitochondrial volume density among treatments (n = 3 for siRNA treatment, and 4 for control). (F) Birds with low *MEF2C* expression showed higher blood glucose levels after 3 h of fasting. (G) Knockdown of *MEF2C* gene decreased glycogen storage in muscle, glucose utilization in exercise, and increased relative activity (n = 5 for siRNA treatment, and 4 for the control group).
(TIF)

S5 Fig Oxygen response in high- and low-altitude tree sparrows. (A) Rufous-necked snow finch had a significant lower survival rate compared to tree sparrows during experimental period of at least 30 days (n = 14 and 20 for snow finches and tree sparrows). (B) Persistent losses of body weight likely caused the high death rate for inadaptation to high temperature (25°C). (C) Hypoxia decreased muscle mass and (D) induced capillarity as well as (E) severe mitophagy in low-altitude tree sparrow but not highlanders (n = 5 each). (F) High-altitude birds in hypoxia had lower glycemia after 3 hours as well as 6 fasting (n = 5 each) but lowlanders only after 3 hours (n = 6 and 5 for normoxia and hypoxia). (G) Hypoxia increased insulin contents in low-altitude tree sparrows (n = 6 and 5 for normoxia and hypoxia). (H) Glycogen contents was higher in hypoxia than normoxia both in high- (n = 5 each) and low-altitude tree sparrows (n = 6 and 4 for normoxia and hypoxia).
(TIF)

S6 Fig Differentiation and regions under selection across altitudinal populations. (A) Genotypes of lowlander and highlander at two *HBB* SNPs. (B) Amino acid alignment of *HBB* across three species and homology model of tree sparrow HbA. Convergent substitutions were shown in red and β80Gly of highland tree sparrow in the EF interhelical loop was convergent with rufous-necked snowfinch and highland house wren(*Troglodytes aedon*, *Tr*. *ae*), whilst β112Ile convergent with rufous-necked snowfinch and band-winged nightjar (*Hydropsalis longirostris*, *Hy*. *lo*). There was no interchain atomic contact between β112Val and α122His, between β112Val and α106Leu at the α1β1 contact surface, while β112Ile had an additional carbon atomrelative to Val, a van der Waals interaction was formed between β112Val and α122His, between β112Val and α106Leu. (C) Manhattan plot: Outcome fixation index (Fst) for SNPs. (D) Genome-wide association analysis between body weight and the genotypes. QQ-plot showed variation of muscle mass (E) and body weight (F), respectively. (G) greater body weight (R = 0.84 and 0.75, respectively, n = 22).
(TIF)

S7 Fig The full gels for western blots. The bands in black box were nonspecific bands and in red box were target protein.
(TIF)

S1 Table Body mass and RMR for *Pa*.*mo* (L), *Pa*.*mo* (H) and *Py*.*ru*. (DOC)

S2 Table Influences of body mass (g) and species (*Pa*.*mo* (L), *Pa*.*mo* (H) and *Py*.*ru*; ANCOVA with mass as a covariate) on RMR (mLO_2_/h). (DOC)

S3 Table Body mass, RMR and EMR and for different treatments. (DOC)

S4 Table Influences of body mass (g) and RNAi treatment (siMEF2C-EPAS1, siEPAS1 and hypoxia control; ANCOVA with mass as a covariate) on RMR (mLO_2_/h). (DOC)

S5 Table Influences of body mass (g) and treatment (normoxia *Pa*.*mo* (L), hypoxia *Pa*.*mo* (L) and hypoxia *Pa*.*mo* (H); ANCOVA with mass as a covariate) on RMR (mLO_2_/h). (DOC)

S6 Table Influences of body mass (g) and treatment (hypoxia *Pa*.*mo* (L) and normoxia *Pa*.*mo* (L); ANCOVA with mass as a covariate) on RMR (mLO_2_/h). (DOC)

S1 Data Body mass of three Passeridae species from the National Zoological Museum, Institute of Zoology, Chinese Academy of Sciences. (XLSX)

S2 Data Differentially expressed genes (DEGs) across altitudinal songbirds. (XLSX)

S3 Data The hub genes in some trait-related modules at the *Pa*.*mo* (L)—*Pa*.*mo* (H), *Pa*.*mo* (L)—*Py*.*ru* and *Pa*.*mo* (L)—*On*.*ta* levels. (XLSX)
