# Supplementary figures and images for: Physiological and genetic convergence supports hypoxia resistance in high-altitude songbirds
Source: PLoS Genet. 2020 Dec 28;16(12):e1009270. doi: 10.1371/journal.pgen.1009270 (PMC7793309; doi:10.1371/journal.pgen.1009270)

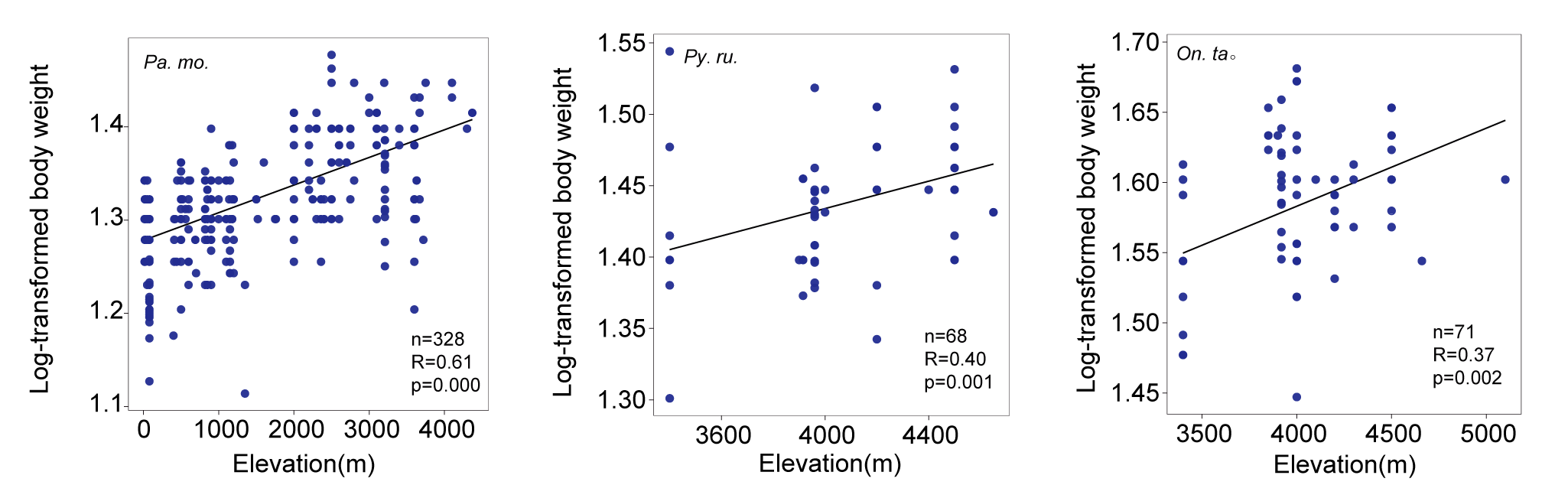

Supplement: S1 Fig — The relationship between log10 body weight (g) mass and elevation (meters) in three songbirds. (TIF) [file pgen.1009270.s001.tif]

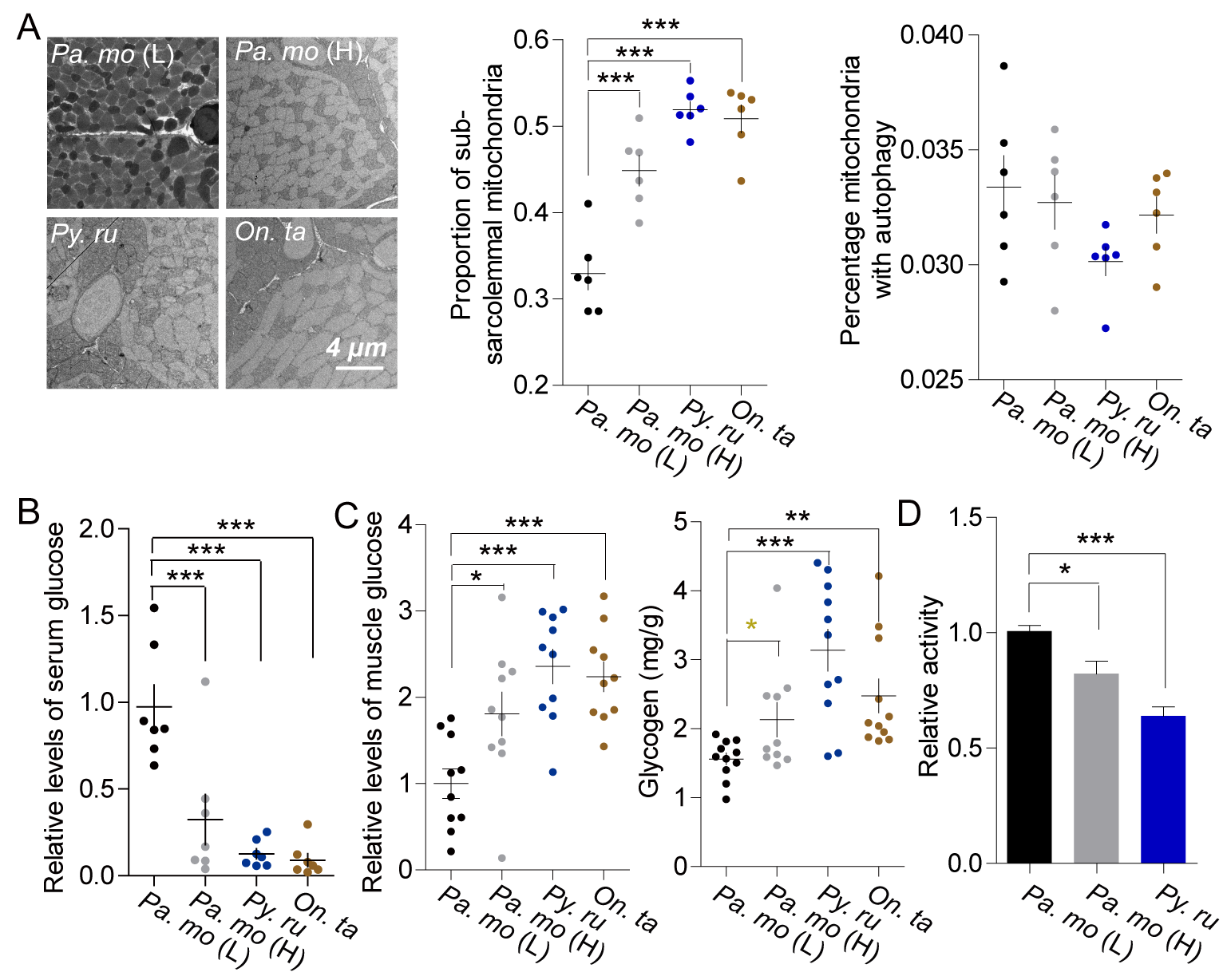

Supplement: S2 Fig — (A) TEM images of mitochondria showed an increase in proportion of subsarcolemmal mitochondria but no mitophagy in high-altitude birds (n = 6 each). (B) Fasting plasma glucose was lower in highlanders compared to lowlanders (n = 7 each). Relative content is the ratio of the peak area of metabolic intermediate to the peak area of the internal standard (heptadecanoic acid). (C) Relative levels of glucose and glycogen contents were increased in highlanders (n = 10 each). (D) Physical activity levels in high and low altitude birds (n = 8 for lowland tree sparrow and rufous-necked snowfinch, and 6 for highland tree sparrow). (TIF) [file pgen.1009270.s002.tif]

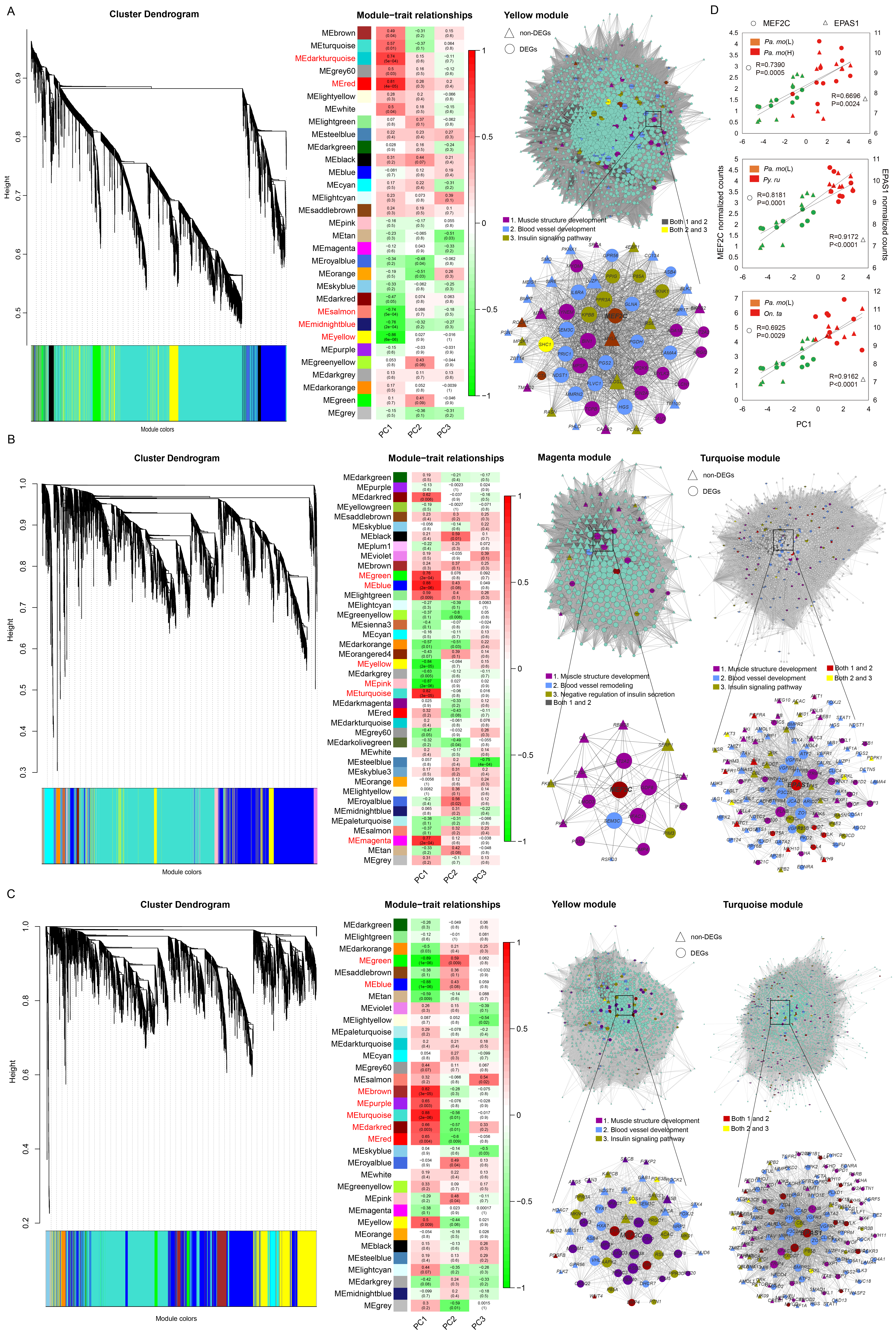

Supplement: S3 Fig — (A) Five modules were identified in Pa.mo (L)—Pa.mo (H) level using WGCNA analysis. MEF2C was identified as a hub gene in yellow module. (B) Six modules were identified in Pa.mo (L)—Py. ru level using WGCNA analysis. MEF2C and EPAS1 were identified as the hub genes in magenta module and turquoise module, respectively. (C) Seven modules were identified in Pa.mo (L)—Ta. on level using WGCNA analysis. EPAS1 were identified as a hub gene in turquoise module. MEF2C was also a hub genes in yellow module, although the correlation coefficient was 0.50 (p value < 0.01). (D) MEF2C and EPAS1 were significant correlated with PC1 of muscle phenotype. (TIF) [file pgen.1009270.s003.tif]

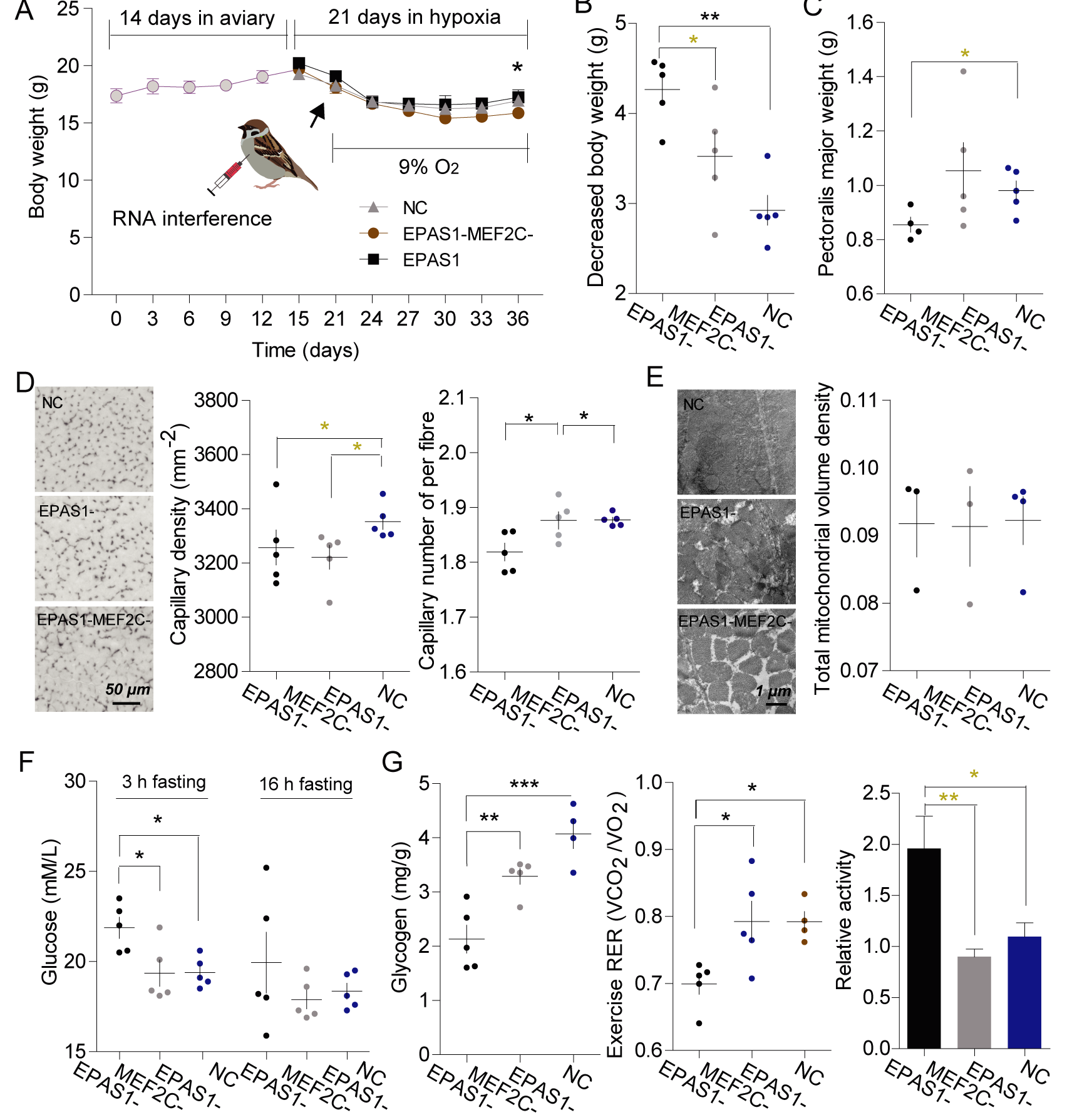

Supplement: S4 Fig — (A) Body weight variation of different siRNA treatments under chronic hypoxia (n = 5 each). (B) Birds with less MEF2C expression showed a dramatic decrease in body weight and had significant increase in loss of body mass than other group (n = 5 each). (C) The loss of muscle mass contributed to reduction of body weight in knockdown of MEF2C (n = 5 each). (D) knockdown of EPAS1 prevented angiogensis in chronic hypoxia (n = 5 each). (E) No difference in total mitochondrial volume density among treatments (n = 3 for siRNA treatment, and 4 for control). (F) Birds with low MEF2C expression showed higher blood glucose levels after 3 h of fasting. (G) Knockdown of MEF2C gene decreased glycogen storage in muscle, glucose utilization in exercise, and increased relative activity (n = 5 for siRNA treatment, and 4 for the control group). (TIF) [file pgen.1009270.s004.tif]

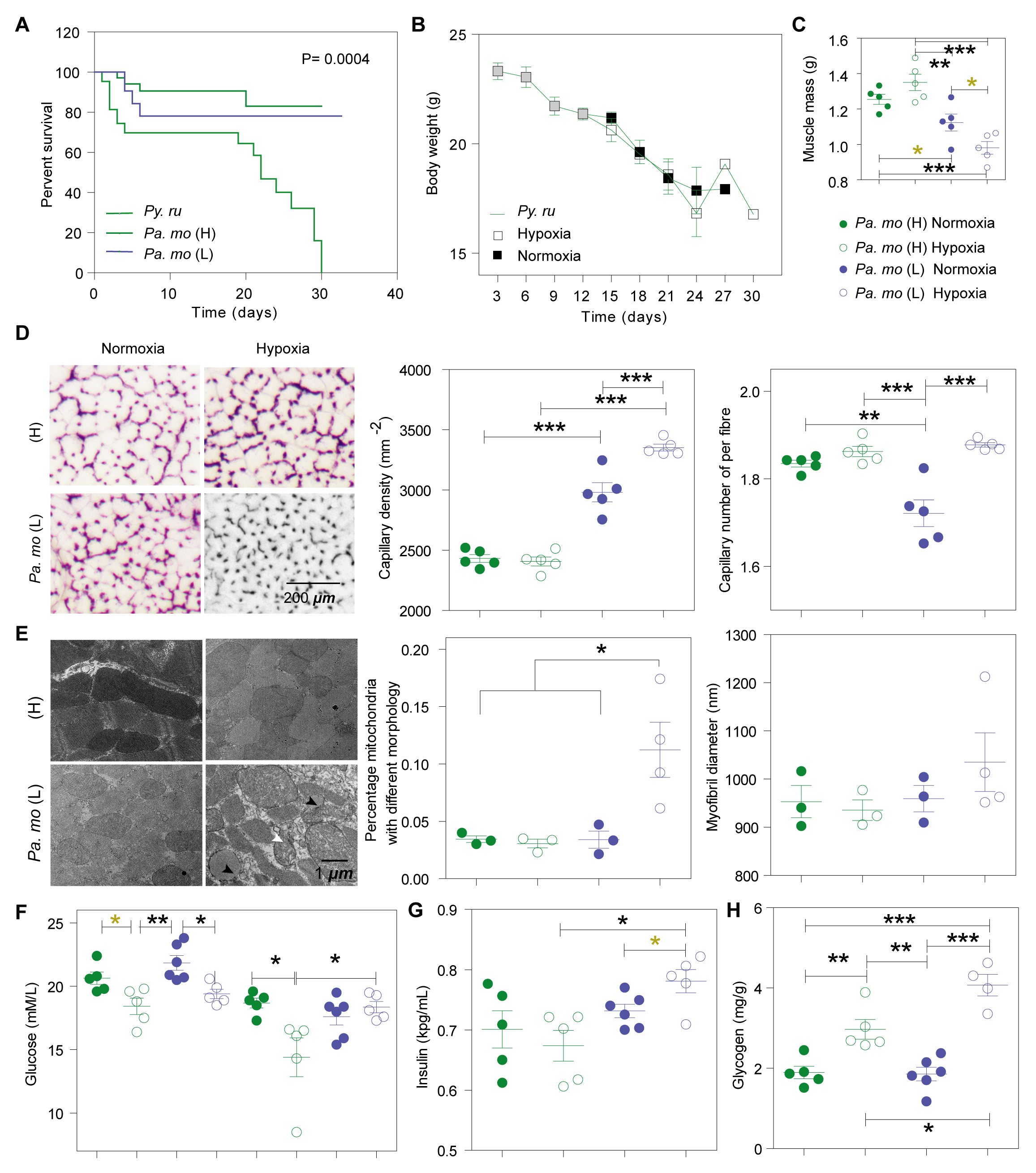

Supplement: S5 Fig — (A) Rufous-necked snow finch had a significant lower survival rate compared to tree sparrows during experimental period of at least 30 days (n = 14 and 20 for snow finches and tree sparrows). (B) Persistent losses of body weight likely caused the high death rate for inadaptation to high temperature (25°C). (C) Hypoxia decreased muscle mass and (D) induced capillarity as well as (E) severe mitophagy in low-altitude tree sparrow but not highlanders (n = 5 each). (F) High-altitude birds in hypoxia had lower glycemia after 3 hours as well as 6 fasting (n = 5 each) but lowlanders only after 3 hours (n = 6 and 5 for normoxia and hypoxia). (G) Hypoxia increased insulin contents in low-altitude tree sparrows (n = 6 and 5 for normoxia and hypoxia). (H) Glycogen contents was higher in hypoxia than normoxia both in high- (n = 5 each) and low-altitude tree sparrows (n = 6 and 4 for normoxia and hypoxia). (TIF) [file pgen.1009270.s005.tif]

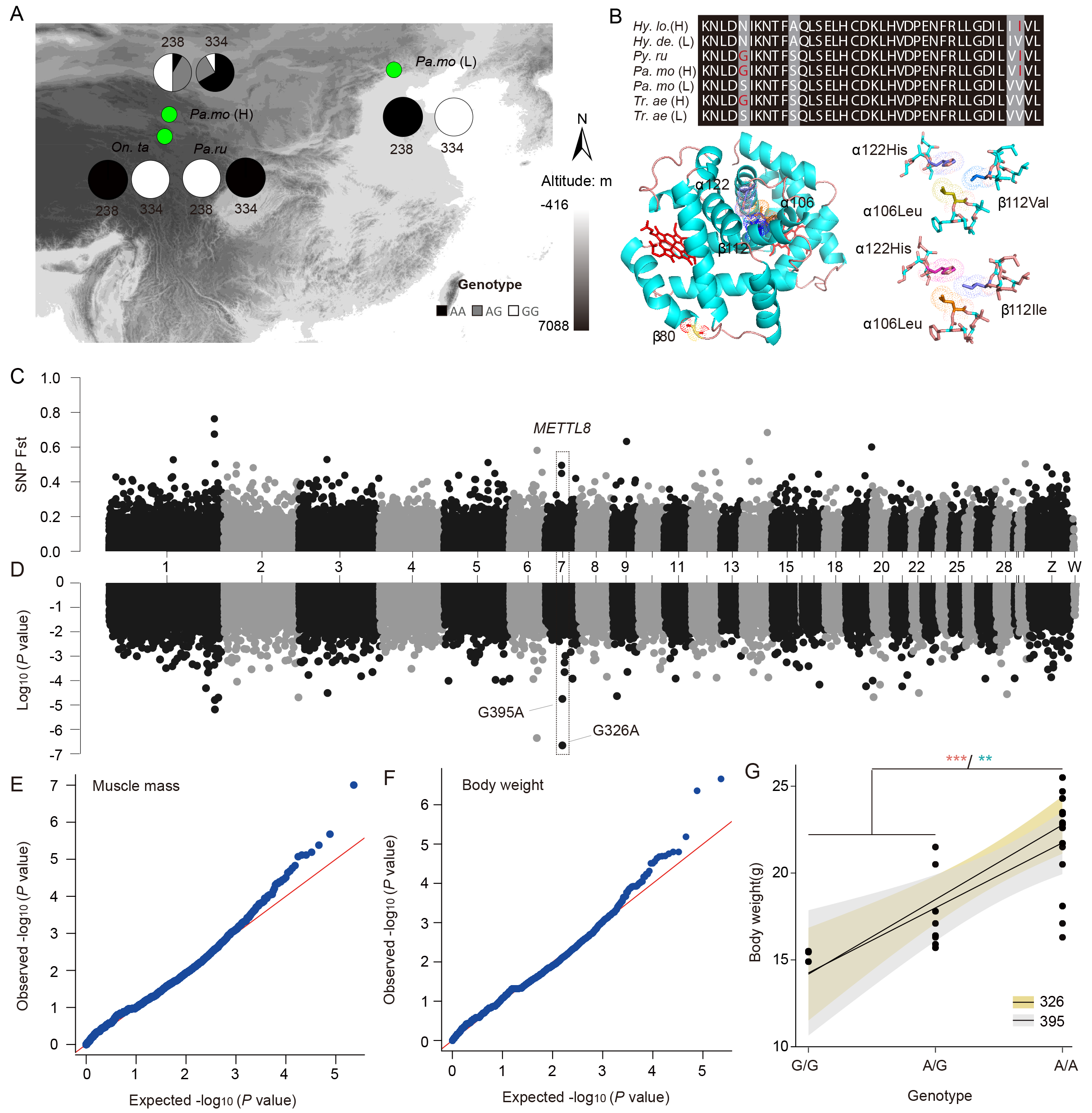

Supplement: S6 Fig — (A) Genotypes of lowlander and highlander at two HBB SNPs. (B) Amino acid alignment of HBB across three species and homology model of tree sparrow HbA. Convergent substitutions were shown in red and β80Gly of highland tree sparrow in the EF interhelical loop was convergent with rufous-necked snowfinch and highland house wren(Troglodytes aedon, Tr. ae), whilst β112Ile convergent with rufous-necked snowfinch and band-winged nightjar (Hydropsalis longirostris, Hy. lo). There was no interchain atomic contact between β112Val and α122His, between β112Val and α106Leu at the α1β1 contact surface, while β112Ile had an additional carbon atomrelative to Val, a van der Waals interaction was formed between β112Val and α122His, between β112Val and α106Leu. (C) Manhattan plot: Outcome fixation index (Fst) for SNPs. (D) Genome-wide association analysis between body weight and the genotypes. QQ-plot showed variation of muscle mass (E) and body weight (F), respectively. (G) greater body weight (R = 0.84 and 0.75, respectively, n = 22). (TIF) [file pgen.1009270.s006.tif]

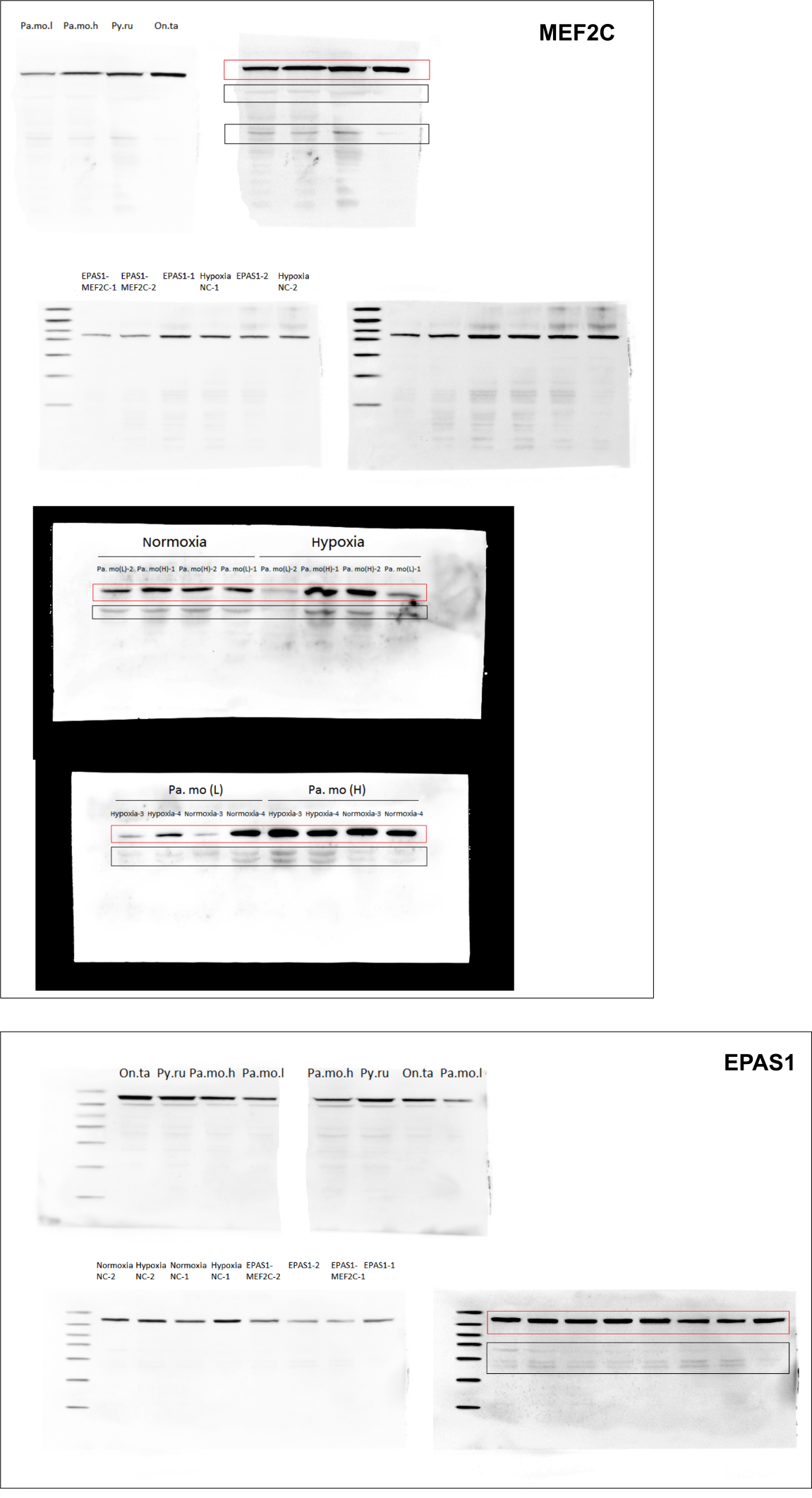

Supplement: S7 Fig — The bands in black box were nonspecific bands and in red box were target protein. (TIF) [file pgen.1009270.s007.tif]
