# Supplementary material for: Physiological and genetic convergence supports hypoxia resistance in high-altitude songbirds
Source: PLoS Genet. 2020 Dec 28;16(12):e1009270. doi: 10.1371/journal.pgen.1009270 (PMC7793309; doi:10.1371/journal.pgen.1009270)
Supplement: S1 Table — (DOC) [file pgen.1009270.s008.doc]

**S1 Table Body mass and RMR for *Pa.mo* (L), *Pa.mo* (H) and *Py.ru***

| **Species** | **Body mass** | **RMR (mLO_2_/h)** |
| --- | --- | --- |
| *Pa.mo* (L) | 17.66 ± 0.24 | 88.94 ± 3.13 |
| *Pa.mo* (H) | 21.35 ± 0.19 | 85.46 ± 4.75 |
| *Py.ru* | 21.49 ± 0.43 | 84.74 ± 3.86 |

Data are presented as means ± SEM. RMR are not corrected for differences in body mass.
