# Supplementary material for: Physiological and genetic convergence supports hypoxia resistance in high-altitude songbirds
Source: PLoS Genet. 2020 Dec 28;16(12):e1009270. doi: 10.1371/journal.pgen.1009270 (PMC7793309; doi:10.1371/journal.pgen.1009270)
Supplement: S2 Table — (DOC) [file pgen.1009270.s009.doc]

**S2 Table Influences of body mass (g) and species (*Pa.mo* (L), *Pa.mo* (H) and *Py.ru*; ANCOVA with mass as a covariate) on RMR (mLO_2_/h)**

| **Source** | **Type III Sum of Squares** | **df** | **Mean Square** | ***F*** | ***P*** |
| --- | --- | --- | --- | --- | --- |
| Corrected model | 0.199^a^ | 3 | 0.066 | 3.396 | 0.042 |
| Intercept | 0.023 | 1 | 0.023 | 1.166 | 0.295 |
| Body mass | 0.177 | 1 | 0.177 | 9.082 | 0.008 |
| Species | 0.198 | 2 | 0.099 | 5.055 | 0.019 |
| Error | 0.332 | 17 | 0.020 |  |  |
| Total | 44.188 | 21 |  |  |  |
| Corrected total | 0.531 | 20 |  |  |  |
| a. R squared = 0.375 (adjusted R squared = 0.264) | | | | | |
