# Supplementary material for: Physiological and genetic convergence supports hypoxia resistance in high-altitude songbirds
Source: PLoS Genet. 2020 Dec 28;16(12):e1009270. doi: 10.1371/journal.pgen.1009270 (PMC7793309; doi:10.1371/journal.pgen.1009270)
Supplement: S3 Table — (DOC) [file pgen.1009270.s010.doc]

**S3 Table Body mass, RMR and EMR and for different treatments**

| **Treatment** | **Body mass** | **RMR (mLO_2_/h)** | **EMR (mLO_2_/h)** |
| --- | --- | --- | --- |
| *Pa.mo* (L) |  |  |  |
| Normoxia | 17.55 ± 0.25 | 79.44 ± 3.97 | 247.8 ± 21.63 |
| Hypoxia | 16.67 ± 0.23 | 80.45 ± 2.45 | 218.7 ± 21.95 |
| siEPAS1 | 16.61 ± 0.30 | 75.95 ± 4.33 | 251.5 ± 25.84 |
| SiMEF2C-EPAS1 | 15.89 ± 0.45 | 108.7 ± 11.80 | 230.2 ± 15.47 |
| *Pa.mo* (H) |  |  |  |
| Normoxia | 18.29 ± 0.34 | 77.07 ± 6.24 | 211.2 ± 8.45 |
| Hypoxia | 19.24 ± 0.41 | 72.44 ± 3.65 | 212.2 ± 12.0 |

Data are presented as means ± SEM. RMR are not corrected for differences in body mass.
