# Supplementary material for: Physiological and genetic convergence supports hypoxia resistance in high-altitude songbirds
Source: PLoS Genet. 2020 Dec 28;16(12):e1009270. doi: 10.1371/journal.pgen.1009270 (PMC7793309; doi:10.1371/journal.pgen.1009270)
Supplement: S4 Table — (DOC) [file pgen.1009270.s011.doc]

**S4 Table Influences of body mass (g) and RNAi treatment (siMEF2C-EPAS1, siEPAS1 and hypoxia control ; ANCOVA with mass as a covariate) on RMR (mLO_2_/h)**

| **Source** | **Type III Sum of Squares** | **df** | **Mean Square** | ***F*** | ***P*** |
| --- | --- | --- | --- | --- | --- |
| Corrected model | 2775.192^a^ | 3 | 925.064 | 4.772 | 0.026 |
| Intercept | 107.084 | 1 | 107.084 | 0.552 | 0.474 |
| Body mass | 569.845 | 1 | 569.845 | 2.940 | 0.117 |
| Treatment | 2775.104 | 2 | 1387.552 | 7.158 | 0.012 |
| Error | 1938.345 | 10 | 193.834 |  |  |
| Total | 111183.185 | 14 |  |  |  |
| Corrected total | 4713.537 | 13 |  |  |  |
| a. R squared = 0.589 (adjusted R squared = 0 .465) | | | | | |
