# Supplementary material for: Physiological and genetic convergence supports hypoxia resistance in high-altitude songbirds
Source: PLoS Genet. 2020 Dec 28;16(12):e1009270. doi: 10.1371/journal.pgen.1009270 (PMC7793309; doi:10.1371/journal.pgen.1009270)
Supplement: S5 Table — (DOC) [file pgen.1009270.s012.doc]

**S5 Table Influences of body mass (g) and treatment (normoxia *Pa.mo* (L), hypoxia *Pa.mo* (L) and hypoxia *Pa.mo* (H) ; ANCOVA with mass as a covariate) on RMR (mLO_2_/h)**

| **Source** | **Type III Sum of Squares** | **df** | **Mean Square** | ***F*** | ***P*** |
| --- | --- | --- | --- | --- | --- |
| Corrected model | 641.743^a^ | 3 | 213.914 | 6.638 | 0.008 |
| Intercept | 116.994 | 1 | 116.994 | 3.631 | 0.083 |
| Body mass | 456.368 | 1 | 456.368 | 14.163 | 0.003 |
| Treatment | 641.743 | 2 | 320.872 | 9.958 | 0.003 |
| Error | 354.458 | 11 | 32.223 |  |  |
| Total | 90805.416 | 15 |  |  |  |
| Corrected total | 996.202 | 14 |  |  |  |
| a. R squared = 0.644 (adjusted R squared = 0.747) | | | | | |
