# Supplementary material for: Physiological and genetic convergence supports hypoxia resistance in high-altitude songbirds
Source: PLoS Genet. 2020 Dec 28;16(12):e1009270. doi: 10.1371/journal.pgen.1009270 (PMC7793309; doi:10.1371/journal.pgen.1009270)
Supplement: S6 Table — (DOC) [file pgen.1009270.s013.doc]

**S6 Table Influences of body mass (g) and treatment (hypoxia *Pa.mo* (L) and normoxia *Pa.mo* (L) ; ANCOVA with mass as a covariate) on RMR (mLO_2_/h)**

| **Source** | **Type III Sum of Squares** | **df** | **Mean Square** | ***F*** | ***P*** |
| --- | --- | --- | --- | --- | --- |
| Corrected model | 329.582^a^ | 2 | 164.791 | 5.326 | 0.039 |
| Intercept | 114.825 | 1 | 114.825 | 3.711 | 0.095 |
| Body mass | 327.154 | 1 | 327.154 | 10.574 | 0.014 |
| Treatment | 177.576 | 1 | 177.576 | 5.740 | 0.048 |
| Error | 216.573 | 7 | 30.939 |  |  |
| Total | 64301.563 | 10 |  |  |  |
| Corrected total | 546.155 | 9 |  |  |  |
| a. R squared = 0.603 (adjusted R squared = 0 .490) | | | | | |
